# Copper Phosphonate Lamella Intermediates Control the Shape of Colloidal Copper Nanocrystals

**DOI:** 10.1021/jacs.2c03489

**Published:** 2022-06-30

**Authors:** James R. Pankhurst, Laia Castilla-Amorós, Dragos C. Stoian, Jan Vavra, Valeria Mantella, Petru P. Albertini, Raffaella Buonsanti

**Affiliations:** † Laboratory of Nanochemistry for Energy (LNCE), Institute of Chemical Sciences and Engineering (ISIC), École Polytechnique Fédérale de Lausanne (EPFL), Rue de l’Industrie 17, Sion 1950, Switzerland; ‡ The Swiss-Norwegian Beamlines, European Synchrotron Radiation Facility (ESRF), Grenoble 38000, France

## Abstract

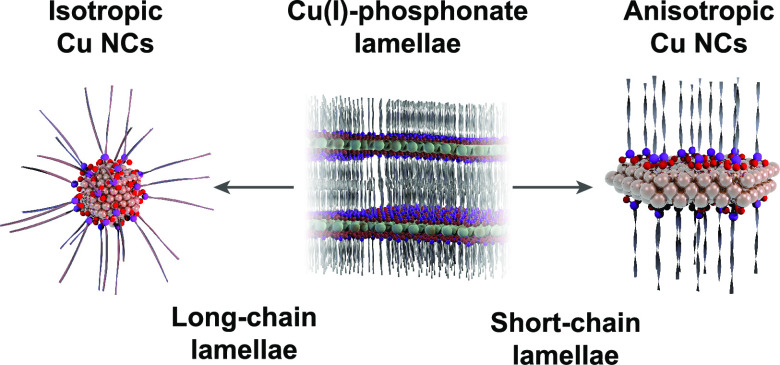

Understanding the structure and behavior of intermediates in chemical reactions is the key to developing greater control over the reaction outcome. This principle is particularly important in the synthesis of metal nanocrystals (NCs), where the reduction, nucleation, and growth of the reaction intermediates will determine the final size and shape of the product. The shape of metal NCs plays a major role in determining their catalytic, photochemical, and electronic properties and, thus, the potential applications of the material. In this work, we demonstrate that layered coordination polymers, called lamellae, are reaction intermediates in Cu NC synthesis. Importantly, we discover that the lamella structure can be fine-tuned using organic ligands of different lengths and that these structural changes control the shape of the final NC. Specifically, we show that short-chain phosphonate ligands generate lamellae that are stable enough at the reaction temperature to facilitate the growth of Cu nuclei into anisotropic Cu NCs, being primarily triangular plates. In contrast, lamellae formed from long-chain ligands lose their structure and form spherical Cu NCs. The synthetic approach presented here provides a versatile tool for the future development of metal NCs, including other anisotropic structures.

## Introduction

Well-defined metal nanocrystals (NCs) offer exciting opportunities as active and functional materials in catalytic, biological, energy conversion, and electronic applications.^1−5^ Their size and shape modulate their properties; therefore, tuning these features during their synthesis is of crucial importance.^1−5^ Colloidal chemistry offers a greater level of precision and tunability along with easy processability in comparison with other synthesis methods, including those based on high-vacuum deposition techniques.^6−8^ Generally speaking, in colloidal synthesis, metal precursors are mixed in a carrier solvent, often with a reducing agent, in the presence of surfactants or ligands that stabilize the final NC product.^6−8^ By fine-tuning several reaction parameters, including temperature, time, atmosphere, and rate of addition of a reagent, the size and shape of the final product can be controlled.^6−8^

Classical nucleation theory describes a simplified process where the metal precursors convert directly into metal nuclei.^9^ However, recent research highlights that the formation of NCs is often more complex.^10^ The realization that NC formation is a multistep process, which includes the formation of metastable prenucleation intermediates, opens up a greater number of possibilities to influence the reaction.^11^ Specifically, the manipulation of these intermediates emerges as a strategy to control the size and shape of the final NC products.

Ligands carry out a number of roles in colloidal synthesis and drastically influence its outcome.^8,12−15^ First of all, the dynamicity and strength of the ligand binding to specific crystal facets determines the rate of growth at that site and, thus, the final NC shape.^16,17^ Second, the ligands can interact with the metal precursor itself to form reaction intermediates.^11^ These intermediates span from simple molecular complexes and clusters to more complex superstructures.^11^ In the former, the coordination chemistry between the metal precursor and the ligands will impact the kinetics of nucleation and growth and, thus, the final NC size and shape.^18−21^ Insight into metal–ligand interactions in precursor complexes has been used to obtain unprecedented shape control of Cu NCs and superior size monodispersity of InP NCs.^22,23^ Coordination polymer lamellae are one example of reaction intermediates with a more complex superstructure. They are layered structures built from the self-assembly of organic ligands and metal ions.^24^ The layering of the coordination polymers is promoted by the intermolecular interactions between hydrocarbon tails in a similar way to lipid bilayers.^25−27^ The coordinating functional group in the ligand, such as a sulfonate, phosphonate, thiolate, or amine, is responsible for bridging multiple metal ions and forming the coordination polymer in either one or two dimensions.^28−31^ Lamellar structures incorporating metal ions or clusters have been isolated as reaction intermediates of colloidal semiconductor NCs and have enabled the synthesis of new complex architectures, including low-dimensional structures, by acting as soft templates during the growth.^32−40^ A few promising examples of lamellar structures acting as growth-directing agents exist for metal NCs, although in a much less advanced stage of development.^30,31^ Generally, despite the growing interest and effort by scientists working in the field, the relationships between the chemical nature of the ligands, the structure of the reaction intermediates, and the final NC products remain to be discovered.

In this work, we contribute to advancing the current knowledge of reaction intermediates in the colloidal synthesis of NCs and the manipulation of these intermediates to target NC products with a desired shape. Specifically, we explore how different phosphonic acid ligands can be used to control the structure of lamella intermediates and how these different structures influence the nucleation and growth in the synthesis of Cu NCs.

Cu NCs have been chosen as a model system as they are promising catalysts for the electrochemical CO_2_ reduction reaction (CO_2_RR).^41−49^ Recent research highlights the intimate relationship between the crystallographic facets exposed by the NC catalyst and the reactivity, even beyond the CO_2_RR.^43,46,50−55^ These studies encourage the continued development of Cu NC shape control, which is still limited compared to what has been achieved for other metals.^7^ Only further insight into their synthesis can drive the current state of research forward.

Herein, we combine in situ X-ray absorption and diffraction techniques with ex situ electron microscopy to understand the chemical and structural changes that occur during NC formation involving lamella intermediates. We highlight the significance of this approach by demonstrating that a structural modification of the reaction intermediates, driven by the chemical identity of the ligands, drastically alters the shape of the NC product, in this case switching from isotropic Cu NCs (spheres) to anisotropic Cu NCs (primarily triangular plates). By providing insight into how lamella intermediates can be rationally controlled to target desired NC products, this study contributes to the development of synthetic schemes for metal NCs beyond trial-and-error approaches.

## Results and Discussion

**Figure 1:**
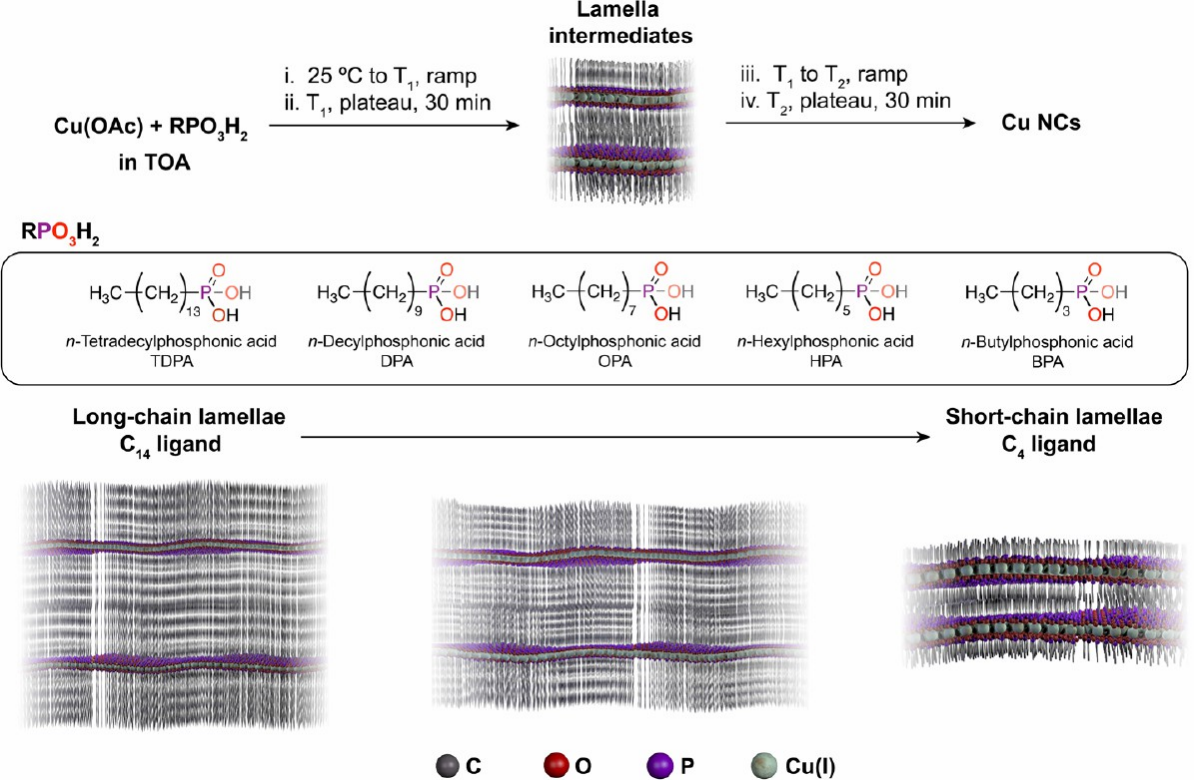
Overview of the synthesis of Cu NCs, using phosphonic acid ligands to build lamella intermediates. *T*_1_ is the lamella formation temperature (typically 150–180 °C); *T*_2_ is the reaction temperature where Cu NCs are formed (greater than 180 °C). The different phosphonic acids used in this work are shown in the box. A schematic representation of lamella structures with decreasing interlayer spacings is sketched at the bottom.

The synthesis of Cu NCs via lamella intermediates that we have developed uses copper(I) acetate (Cu(OAc)) as the copper precursor, an aliphatic phosphonic acid ligand, and tri-*n*-octylamine (TOA) as the solvent and reducing agent (Figure 1). The reaction comprises four stages (Figure S1): (i) a heating ramp from near room temperature to the lamella formation temperature, *T*_1_; (ii) a plateau at *T*_1_ for 30 min; (iii) a heating ramp from *T*_1_ to the reaction temperature, *T*_2_; and (iv) a plateau at *T*_2_ for 30 min. This approach was inspired by the recent discovery that spherical Cu NCs form from copper phosphonate lamellae when Cu(OAc) reacts with *n*-tetradecylphosphonic acid (TDPA) in TOA at 270 °C.^31^ In that study, the long-chain lamellae collapse into reverse micelles during heating, resulting in a size-focusing effect for the Cu spheres.^31^ Instead, to make use of the lamellae as a shape-directing agent, the lamella structure must remain intact during the NC nucleation and growth phase.^32−40^ Tuning the length of the aliphatic chain should have an important effect on the thermal stability and reduction behavior of the lamellae. However, predicting the structure of the lamellae and their thermal stability from the chemical identity of the ligands is not trivial. Multiple scenarios are indeed possible. For example, long-chain lamellae are expected to be more thermally robust than short-chain lamellae if the aliphatic chains are fully interdigitated as more van der Waals interactions would exist between neighboring chains.^56−58^ The opposite is anticipated when only partial interdigitation between the aliphatic chains occurs. In that case, the higher structural flexibility of the long-chain lamellae would render them less stable and more prone to entropy-driven dissolution. Experiments are needed to validate one scenario over the other.

Therefore, we investigated several phosphonic acid ligands with different aliphatic tail lengths, which are drawn in Figure 1: *n*-tetradecylphosphonic acid (TDPA, C_14_ tail); *n*-decylphosphonic acid (DPA, C_10_ tail); *n*-octylphosphonic acid (OPA, C_8_ tail); *n*-hexylphosphonic acid (HPA, C_6_ tail); and *n*-butylphosphonic acid (BPA, C_4_ tail).

### Characterization of the Copper Phosphonate Lamella Intermediates

**Figure 2:**
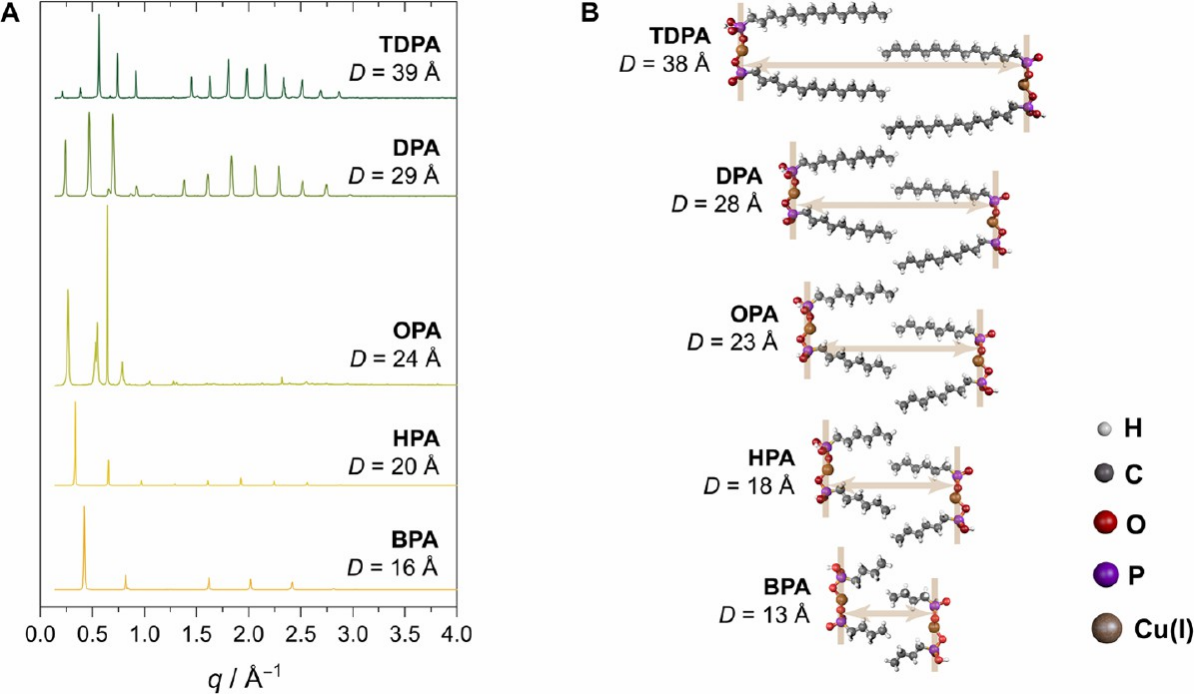
(A) XRD patterns for lamella intermediates with different-length phosphonic acid ligands isolated at 150 °C. The lamella interlayer spacings (*D*) are given for each intermediate. (B) Molecular mechanics models for the different lamellae used to predict the interlayer spacings indicated by brown arrows. Details of the molecular mechanics calculations are given in the Supporting Information.

To verify that all of the ligands generate copper phosphonate lamellae and to learn more about their structure, we first analyzed the intermediates that form at 150 °C by X-ray diffraction (XRD). This temperature was chosen as the Cu-TDPA lamellae were previously detected to form during the heating ramp between 90 and 160 °C.^31^Figure 2A shows that lamellar structures are present in all cases and no diffraction from metallic Cu is observed. The periodic set of low-angle diffraction peaks is characteristic of lamellae and allows the interlayer spacing (*D*) to be determined. The *D* spacings that are derived from these diffraction peaks show that the interlayer distances are governed directly by the length of the phosphonic acid ligands. Indeed, the measured *D* values are in very good agreement with the distances approximated using simple molecular mechanics models of the different Cu(I) phosphonate building blocks (Figure 2B). They match closely with double the length of the Cu phosphonate unit (including the aliphatic tail), indicating that minimal interdigitation of the chains occurs. In addition, the Fourier-transform infrared (FT-IR) absorption spectra confirm that the phosphonic acids are present in the lamella structures as fully deprotonated phosphonate ligands (Figure S2).

### Effect of the Phosphonic Acid Chain Length and the Reaction Temperature on the Morphology of the Reaction Product

**Figure 3:**
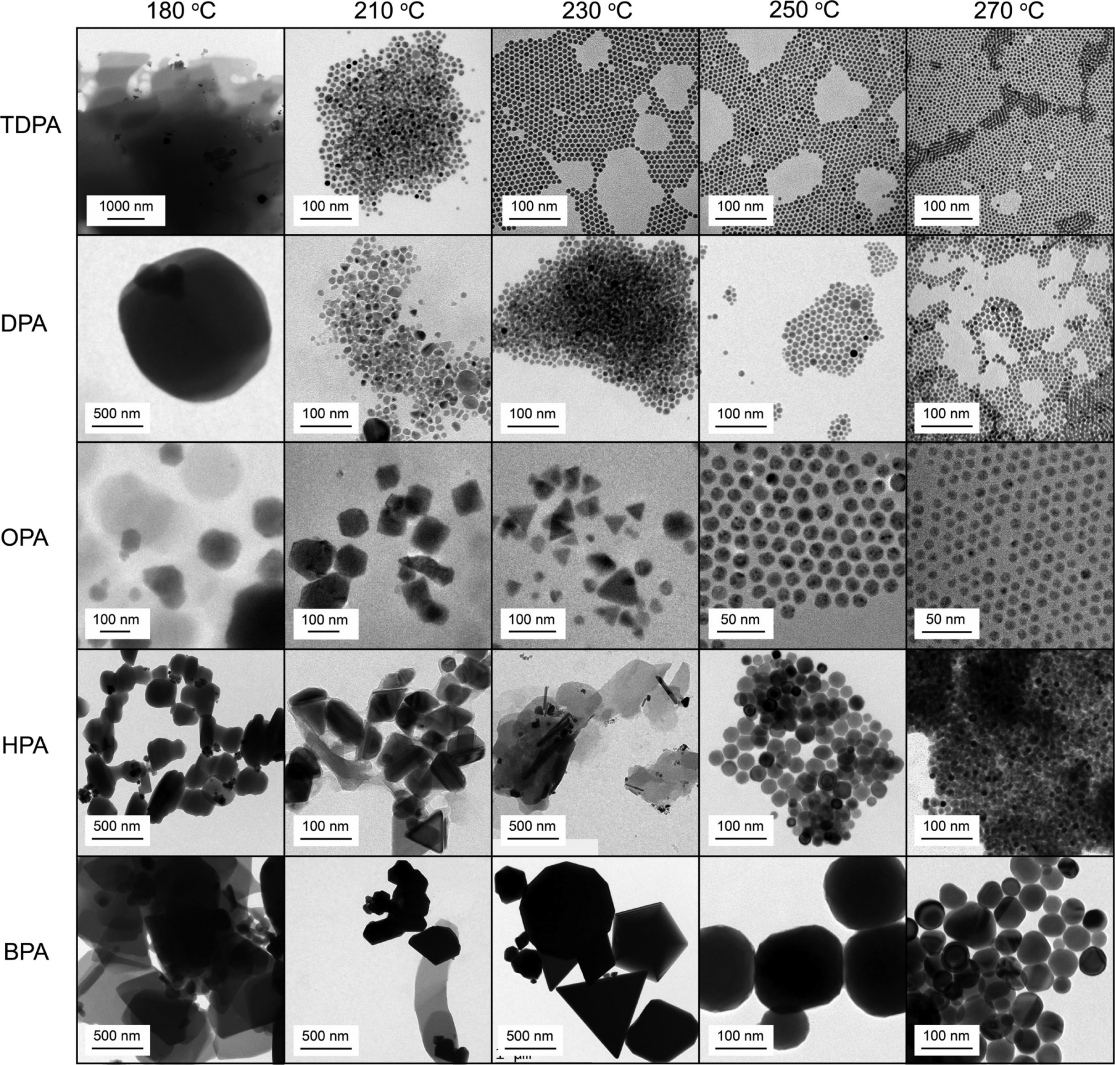
TEM images of the collected products from reactions involving Cu(OAc) and phosphonic acid ligands in TOA solvent, showing the effect of ligand tail length and reaction temperature *T*_2_.

Having assessed that all of the ligands form lamellae, we investigated the products from reactions at different *T*_2_ temperatures, while keeping *T*_1_ at 180 °C, which was chosen based on our previous work with Cu-TDPA.^31^Figure 3 shows representative transmission electron microscopy (TEM) images of these products. At higher temperatures, namely, 250 and 270 °C, spherical Cu NCs form regardless of the aliphatic chain length of the ligands. At intermediate reaction temperatures, between 210 and 230 °C, spherical Cu NCs are obtained with the long-chain phosphonic acid ligands TDPA and DPA, although their size dispersity is generally poorer compared to higher-temperature reactions (Figures S3–S5). Instead, a mixture of triangles and thinner sheets is observed for the short-chain ligands OPA, HPA, and BPA. Finally, at the lowest reaction temperature of 180 °C, very large, nebulous structures are isolated when long ligands (TDPA and DPA) are used. More discrete globules are observed with OPA and HPA, with random networks forming in the latter. The reaction including BPA generates distinctly 2D sheets.

### Monitoring the Chemical and Structural Transformation of the Lamella Intermediates via In Situ XAS and XRD

To understand the chemical and structural changes occurring in the reaction mixture during the synthesis, we performed in situ Cu K-edge X-ray absorption spectroscopy (XAS) and XRD measurements using synchrotron radiation. For these studies, a custom three-neck flask was used, which quite accurately replicates the conditions of a typical synthesis (see Figure S6).

**Figure 4:**
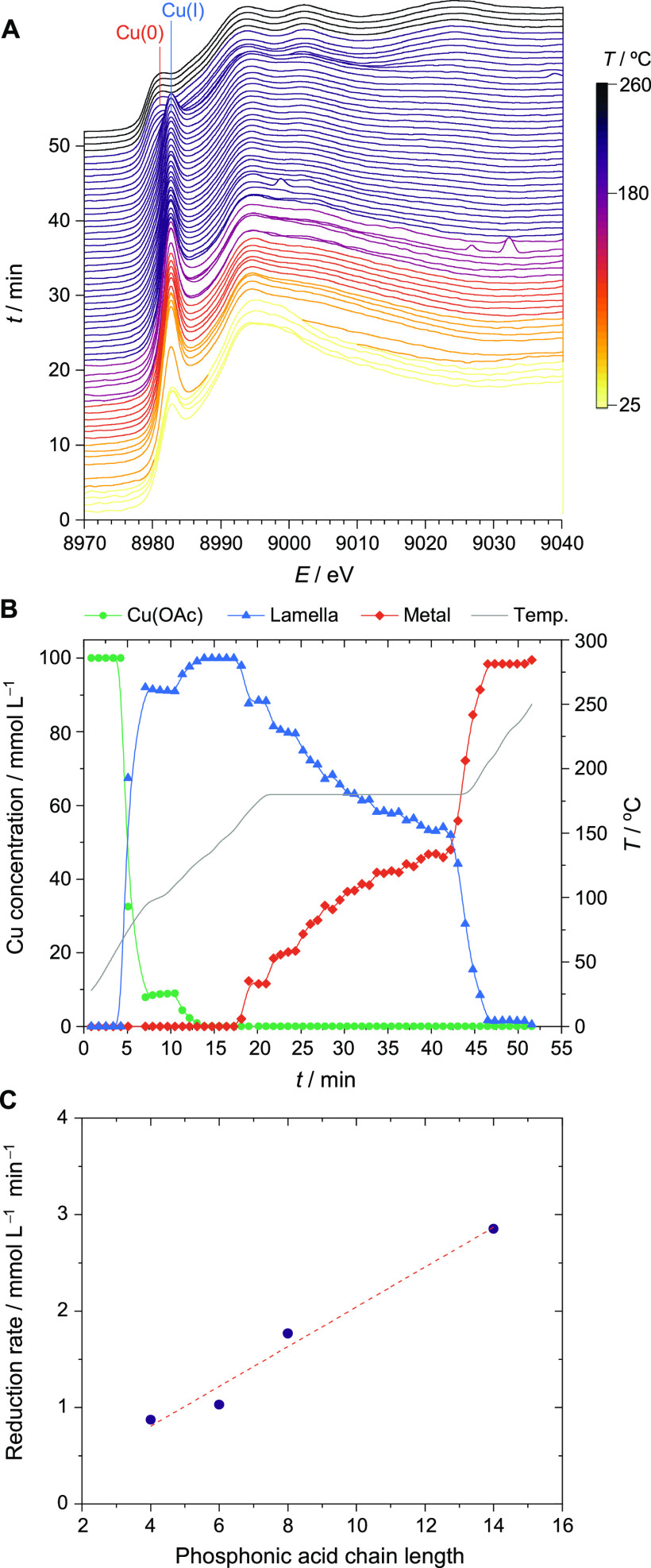
In situ X-ray absorption spectroscopy data, showing (A) XAS spectra plotted as a function of time, revealing the conversion of Cu(OAc) to the lamella intermediate and subsequent reduction to metallic Cu; the spectra are colored according to the reaction temperature. (B) Kinetic profile for the three principal components in the reaction, following the evolution and reduction of the lamella intermediate with time (the reaction temperature is also plotted). Reactions involving TDPA are shown as representative examples. (C) Cu(I) to Cu(0) reduction rate vs the length of the phosphonic acid chain in the lamella structure, showing a linear correlation (*R*^2^ = 96%).

XAS provides information on the Cu oxidation state as well as the coordination geometry and offers insight into how these parameters evolve with time. For these experiments, the reaction temperature *T*_2_ was set at 270 °C, which is the highest limit of the syntheses studied above. The reaction with TDPA will be discussed as a representative example as the data were similar for all of the phosphonic acid ligands (Figures S7–S10). Figure 4A shows that the pre-edge feature of the XAS spectrum becomes very sharp and intense during the first heating ramp to *T*_1_ = 180 °C, which is when the conversion from Cu(OAc) to the lamella intermediates occurs. However, the energy of the peak does not change, indicating that Cu remains in the +1 oxidation state. The dramatic increase in intensity of the pre-edge XAS feature indicates that the coordination geometry in the lamellae is very different from that in the Cu(OAc) starting material. Previous work has attributed this feature to the Cu 1s to 4p electronic transition, which is very sensitive to the coordination geometry and coordination number.^59^ Specifically, a sharp and intense pre-edge feature is assigned to low-coordinate Cu(I) ions, such as a linear, two-coordinate geometry or similar. In a linear geometry, the energy of the Cu 2p_*z*_ orbital rises due to the donated electron density from the two axial ligands, leaving the Cu 2p_*x*,*y*_ non-bonding orbitals lying at lower energy.^60−62^ The result is a very strong absorption due to the transition from the 1s orbital into these low-lying orbitals.

During the second heating ramp to 270 °C, this pre-edge feature reduces in intensity and shifts to lower energy, which corresponds to the reduction from Cu(I) to Cu(0).^60,61^ Specifically, this change indicates the reduction of the lamella intermediates into metallic Cu NCs. Indeed, the final spectra in all cases matched that of pure metallic Cu very well.

Deconvolution of the in situ data by principal component analysis (PCA) provides a more detailed picture of the reaction as each component can be followed as a function of time (Figure 4B and Figures S11–S14). We note that the extracted spectra for the lamella intermediates were similar for reactions involving different-length phosphonic acid ligands in the sense that they revealed identical oxidation states and coordination geometries in each reaction (Figures S15–S18). Figure 4B shows that the concentration of metallic Cu steadily increases during the temperature plateau at 180 °C before rapidly rising during the heating ramp from 180 to 270 °C. The TEM of the lamella sheets isolated after the 180 °C plateau revealed the presence of higher-contrast spots that are consistent with the growth of metallic clusters within or on top of the lamella (Figure S19). Finally, during the second heating ramp toward 270 °C, the remainder of the lamella intermediate rapidly converts into metallic Cu, which is coincident with the visible formation of Cu NCs in the flask via a yellow to brown color change of the solution.

One interesting result emerges from the kinetics plots, which is that the reduction rate from Cu(I) to Cu(0) during the temperature plateau at 180 °C decreases with the chain length of the phosphonic acid ligands, with the slowest reduction rate being measured for BPA (Figure 4C). Inductive electronic effects do not explain the trend in the reduction rate with the ligand chain length as longer carbon chains should stabilize Cu(I) against reduction. Instead, the structural stability of the lamellae, which will be discussed later in the manuscript, might provide a better explanation for it, i.e., the more thermally stable lamella structures retard the reduction of Cu(I) to Cu(0).

**Figure 5:**
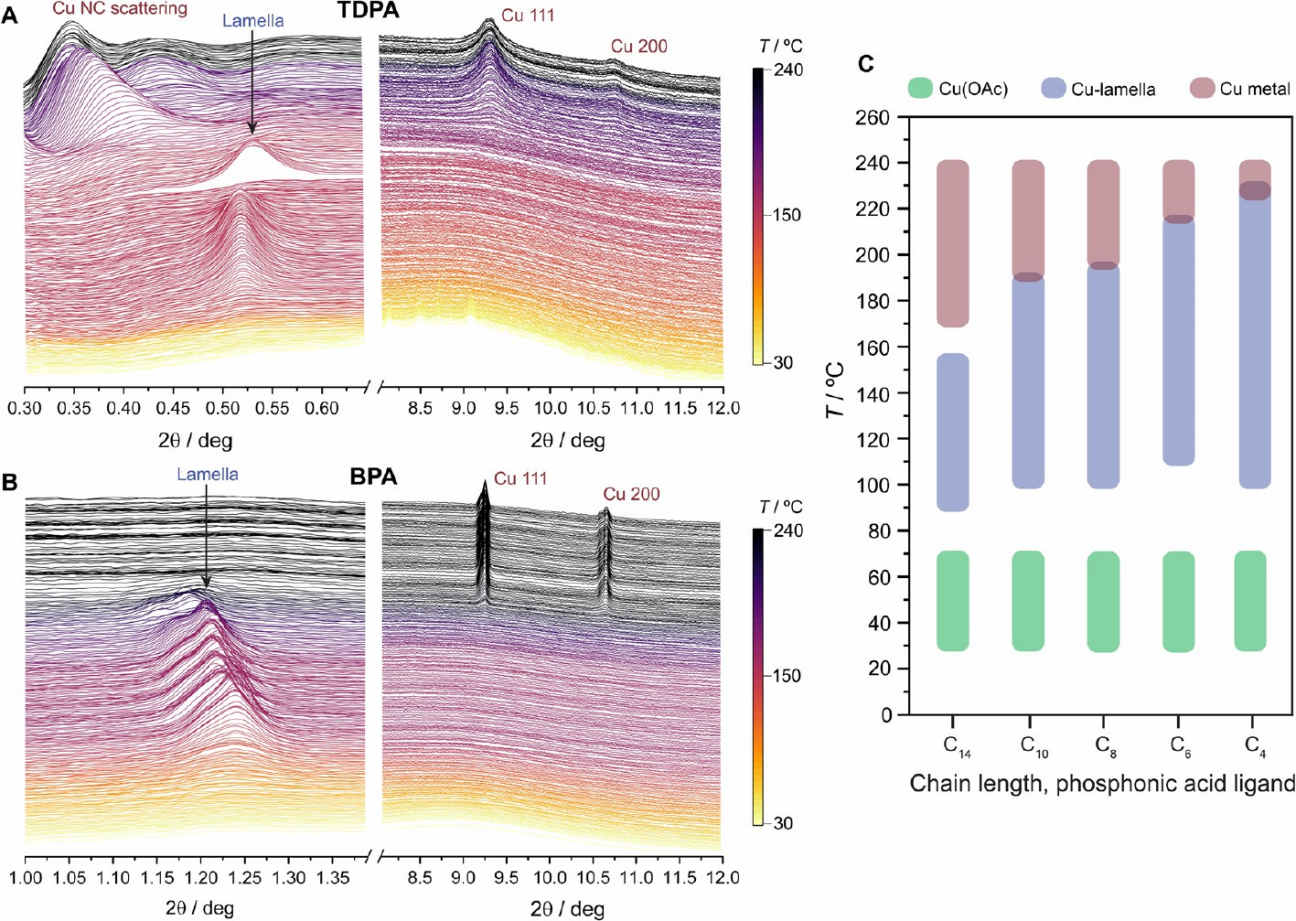
In situ X-ray diffraction data, showing (A) the low-angle and high-angle regions for a reaction containing TDPA, Cu(OAc), and TOA, and (B) the low-angle and high-angle regions for a reaction containing BPA, Cu(OAc), and TOA. Lamella phases and scattering from Cu nuclei and NCs are seen in the low-angle regions, while metallic phases are seen in the high-angle regions (all labeled). Diffractograms are plotted as a function of time, and the reaction temperature is shown as a color map. We highlight that the lamella intermediates are poorly soluble in TOA, leading to some fluctuations in the XRD signal despite stirring, most notably in (A). (C) Summary of the temperatures at which the different phases are observed, revealing the relationship between lamella stability and the phosphonic acid tail length.

In situ XRD experiments provided additional details on the lamella structures and their conversion into crystalline Cu. Representative patterns are shown in Figure 5 for the longest (TDPA) and the shortest (BPA) phosphonic acids (other data are reported in Figures S20–S24). For these experiments, *T*_1_ and *T*_2_ were set to 150 and 240 °C, respectively, to focus on the formation of anisotropic shapes. As a general trend, the lamella phases, which are distinguishable in the low-angle region, rapidly form from Cu(OAc) during the initial heating stages and are stable for the duration of the first temperature plateau at *T*_1_. The corresponding lamella *D* spacings in these in situ XRD experiments are identical to those for the isolated lamellae (Figure 1) and depend on the length of the phosphonic acid ligand (Figure S25). During the second heating ramp to 240 °C, the lamella phases convert into metallic Cu whose characteristic peaks are observed at higher angles, around 9 and 11°.

For the TDPA lamella (Figure 5A), the principal lamella peak at 0.52° suddenly disappears at 180 °C. Following this event, a scattering peak is observed at 0.42°, which rapidly grows in intensity and shifts to even lower angles (minimum 2θ = 0.35°). These changes indicate that the long-chain lamella collapses. The growth and shift of the low-angle scattering peak is accompanied by the growth of a set of very broad peaks for the metallic phase of Cu. The sharply oscillating low-angle scattering pattern observed when the metallic Cu NCs have fully developed is consistent with the formation of small, monodisperse nanoparticles;^63,64^ the product from this reaction are indeed 6 nm monodisperse spheres.

For the BPA lamella (Figure 5B), the principal lamella peak at 1.24° is still observable in the mixture above 230 °C, indicating that the short-chain lamella does not collapse in the same way as the long-chain lamella. Around this same temperature, sharp Cu peaks appear very quickly in the wider-angle region, indicating the rapid formation of larger Cu NCs. The lamella phase is persistent during the Cu NC growth phase, and we also note that the initial lamella peak shifts to lower angles during heating. These changes correspond to a *D*-spacing expansion from the initial 14.36 Å to 15.57 Å at 150 °C and to 16.78 Å at 230 °C. This expansion may evidence the formation of small Cu clusters within the 2D lamella structure. Indeed, quasi-stable Cu clusters in the sub-nanometer regime have been studied,^65,66^ and similar-size clusters could be assembling laterally within the lamella template. With these nuclei supported in the lamella assembly in proximity with one another, their fusion and growth into a larger crystallite is expected to be rapid, in agreement with the rapid growth of the metallic Cu peak in the in situ XRD experiment. In comparison, after the long-chain lamella collapses and the Cu ions are reduced to metallic nuclei, their growth into spherical NCs occurs much more slowly as they are dispersed in solution.

Interestingly, the temperature at which the lamellae transform into crystalline metallic Cu highly depends on the aliphatic tail length of the ligand (Figure 5C and Figure S26). The persistence of the lamella phase in the mixture follows a linear trend between the two extremes discussed above, with shorter-chain phosphonic acid ligands imparting greater stability on the lamella assembly. This increased structural stability slows down the aforementioned Cu(I) to Cu(0) reduction during the first temperature plateau (Figure 4C) and delays the onset of Cu crystallization. The higher stability of the shorter-chain lamellae is in line with the lack of any significant interdigitation among the aliphatic chains (Figure 2).

### Synthesis Optimization toward 2D Cu Nanostructures

The persistence of the lamellae from shorter-chain phosphonic acids at higher temperatures during the Cu NC crystallization is the required condition that enables templating effects for the attainment of 2D structures. The homogeneity of the triangular Cu NCs formed with OPA at 230 °C was decent (Figure 3), although the reaction yield was too low to concentrate the following optimization on those. The Cu NCs obtained from the BPA-derived lamella at 230 °C were promising (Figure 3); nevertheless, they lacked uniformity in size and shape.

Inspired by previous work on lamella templating of 2D CdSe NCs,^33,34,37^ we attempted mild annealing of the BPA lamella at the formation temperature (*T*_1_) to allow the Cu nuclei more time to fuse within the molecular template. However, this strategy was largely unsuccessful as the NC formation requires higher temperatures in this reaction mixture; products from these reactions contained large amounts of residual lamella (Figure S27). Heating the reaction mixture directly to *T*_2_, with either slow or fast heating ramps, also did not help improve the sample uniformity, and a mixture of unreacted lamellae and particles were obtained (Figure S28). These results evidenced the importance of the plateau stage at *T*_1_ for the lamella structure to self-assemble and for enabling the formation of metallic Cu species (i.e., nuclei/seeds) within them. We also suspected that side products or other impurities from the lamella formation step might contribute to the polydispersity in the final product. Therefore, BPA-lamellae were presynthesized, purified by solvent washing and centrifugation, and used as the synthesis precursor. The reaction products were similar to those where the lamellae were generated in situ during the synthesis (Figure S29).

As mentioned above, the yield of metallic Cu NCs in lower-temperature reactions was generally low and large amounts of unreacted lamella were often observed. To circumvent this issue, we attempted to promote the Cu(0) formation at a lower temperature in the lamella host by introducing a reducing agent. However, injection of ascorbic acid into the BPA reaction mixture resulted in the formation of large aggregates (Figure S30), suggesting that the combination of strong reductants with these lamella intermediates disrupts the templating effect.

**Figure 6:**
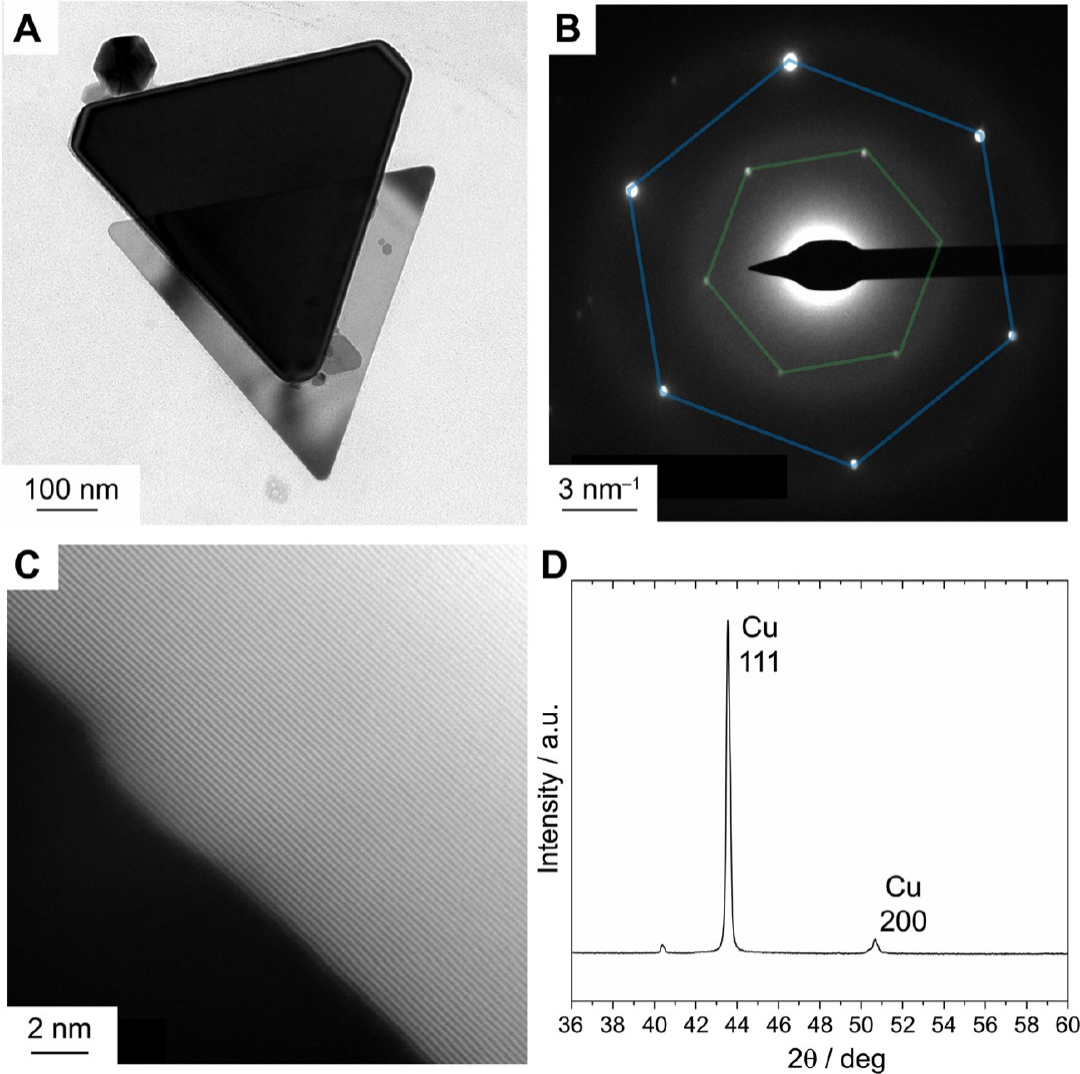
(A) Bright-field TEM image of anisotropic Cu NCs synthesized from lamella templates. (B) SAED image for a single triangular Cu NC, showing the forbidden 1/3(422) reflection (highlighted in green) and the (111) reflection (highlighted in blue). (C) HAADF-STEM image of the edge of a triangular Cu NC. (D) XRD pattern for the anisotropic Cu NCs, showing the preferential orientation of the (111) facet, which arises mainly from triangular Cu NCs.

Another plausible hypothesis to explain the polydispersity in both size and shape of the BPA-derived samples is the poor colloidal stability and solubility of the BPA-lamellae themselves. The stacking of poorly dispersed lamellae could lead to aggregation of the Cu NCs upon decomposition of the lamella structure. We reasoned that improving the colloidal stability and solubility of the lamella intermediates might impart greater homogeneity in the final NC product. Based on our previous experience, we preferred to avoid carboxylic acid ligands as they facilitate copper oxidation during the synthesis. Therefore, we opted for oleylamine (OLAM), which is a common solvent and surfactant in colloidal NC synthesis.^67^ Furthermore, it could act as a mild reductant along with TOA, which is already present. Different OLAM concentrations were screened, and a 1:1 molar ratio of OLAM and Cu(OAc) was found to improve the uniformity of both the lamella intermediate and Cu NC product (Figure S31). Heating a mixture of Cu(OAc), BPA, TOA, and OLAM to 220 °C produced a sample of Cu NCs containing 57% of anisotropic plates, 93% of which are triangles with average edge lengths of 385 nm (Figure 6A and Figures S32 and S33). Without OLAM, anisotropic shapes accounted for only 28% of the sample. The size distribution remains quite broad, yet it is improved after introducing OLAM, while the equilaterality and sharpness of the triangular Cu NCs were almost identical after introducing OLAM (Figures S33 and S34). We highlight that the temperature of 220 °C was chosen as the in situ XRD studies revealed that the lamella structure is persistent and that Cu NC reduction and crystallization can occur here (Figure 5C).

Selected-area electron diffraction (SAED) of a single triangular NC revealed the hexagonal patterns for the (111) and 1/3(422) reflections of the face-centered cubic structure of crystalline metallic Cu (Figure 6B). Note that the latter reflection is formally forbidden but is often observed in thin, 2D metallic NCs that feature stacking faults from non-3*n* layers in the crystal structure that are bound by atomically flat faces.^68−72^ The periodic contrast in the HAADF-STEM image of the edge of a triangular Cu NC corresponds to the (200) planes of metallic Cu with a lattice spacing of 1.9 Å (Figure 6C). Finally, X-ray diffraction evidenced an intense peak for the (111) reflection due to the preferred orientation and high ratio of the (111) facet presented by the triangular Cu NCs (Figure 6D).

Copper nanowires and nanosheets have previously been synthesized through selective capping of the (111) facet;^68,73,74^ thus, OLAM could conceivably be implicated in a similar role. However, only the unique combination of BPA, which forms thermally stable lamellae, and OLAM results in a higher yield of triangular plates. In contrast, when OLAM was employed in combination with TDPA, monodisperse, spherical Cu NCs were still obtained from the reaction (Figure S35), thereby ruling out the anisotropic NC growth by capping of the (111) facet.

Additional in situ XRD measurements evidenced that OLAM is not incorporated into the lamella structure and does not impact the structural transformations during the reaction (Figures S36–S43). TEM characterization of the BPA-lamella intermediates in the presence of OLAM (Figure S44A) revealed that small, spherical Cu crystalline domains are present at 150 °C, which is consistent with the hypothesis of Cu nuclei forming within the lamellae. The most striking difference between the BPA-lamella intermediates formed in the absence and presence of OLAM is that the latter is uniform and well-assembled circular lamella sheets (Figure S44B) and possesses an improved colloidal stability compared to those obtained without OLAM. A structure of the phosphonate lamellae wherein OLAM binds to the low-coordinate surface copper sites is envisioned (Figure S45).

### Mechanistic Insight into the Lamella-Assisted Synthesis of Cu NCs

**Figure 7:**
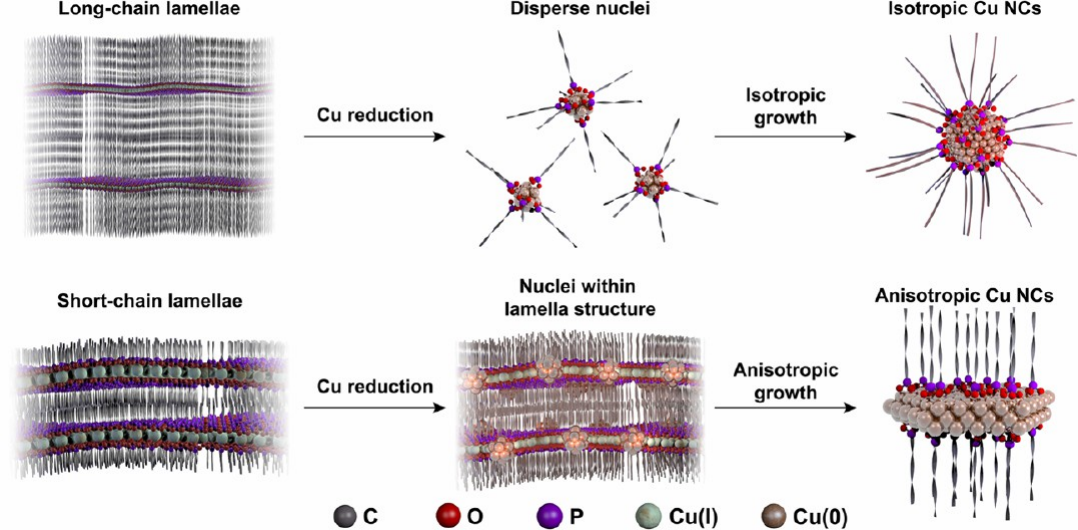
Schematic illustration of lamella-assisted synthesis of Cu NCs for achieving shape control. Short-chain lamellae are thermally stable at the reduction temperature, allowing templated nucleation and leading to anisotropic NC growth.

All the above discussions provide evidence for the mechanistic picture that is illustrated in Figure 7. Copper phosphonate lamellae forming from long-chain phosphonic acids decompose rapidly and release well-dispersed nuclei that go on to form monodisperse spheres. In contrast, short-chain lamellae are more thermally robust than long-chain lamellae; thus, their structure persists during the Cu(I) reduction step into the Cu(0) nuclei and direct the growth of the resulting Cu NCs toward anisotropic shapes. Furthermore, we have learned that improving the dispersibility of the short-chain lamella intermediates in the reaction medium plays an important role in improving the size and shape homogeneity of the final product toward mostly triangular plates. Long-chain primary amines emerged as suitable reagents to realize this condition.

While the presence of the lamella structure explains the mechanism of anisotropic growth, it is unclear why the majority of Cu NCs become triangular shapes. A dominant role of the ligands in determining this final shape via passivation of the (111) surface during the NC growth is unlikely, considering that the anchoring group is the same (i.e., phosphonate) across all samples and different shapes were still obtained. The final shape of metal NCs has previously been connected to seeds of specific shapes forming after nucleation; in this situation, triangular plates should grow from platelet seeds with stacking faults.^75,76^ The initial reduction rate of the metal precursor has been proposed as one of the determining factors for the seed structure.^76^ Specifically, a lower reduction rate of the metal precursor has been correlated to the formation of seeds with twin planes and stacking faults.^76^ In the present study, the slowest reduction rate of Cu(I) to Cu(0) was observed for the BPA-lamella during the first temperature plateau (Figure 4C), which is a crucial step for the synthesis (Figure S28). Thus, we suggest that seeds containing twin planes and stacking faults form under the slow reduction conditions induced by the short-chain lamella, resulting in triangular NCs. Therefore, the interplay of the structure-induced thermal stability of the lamellae and the resulting kinetics of Cu(I) reduction emerges as the most likely explanation for the shape control of Cu NCs achieved using lamella intermediates.

The improved colloidal stability of the BPA lamellae with the addition of OLAM, which leads to a more homogeneous reaction medium, was proven to increase the yield of the triangular plates (Figure S45). The observation that uniform and well-assembled circular lamella sheets form in the presence of this ligand mixture is intriguing. We acknowledge that the shape of the lamella intermediate does not directly translate into the final NC shape. However, a correlation between the two cannot be completely ruled out. We note that similar observations have been made in NC synthesis using reverse micelles, wherein the shape of the micelles was observed to impact the shapes of the resulting NCs, although a detailed atomistic picture is still missing.^77−80^

Follow-up studies are still needed to further understand the evolution of the cooperative organization of inorganic and organic molecular species during the nucleation and growth of the Cu clusters within the lamella framework. These studies are challenging and will require the implementation of other techniques, which include in situ total scattering experiments^81^ and nuclear magnetic resonance spectroscopy.^82^ Yet, these in-situ investigations are important to ascertain whether the Cu clusters truly grow within the layers of the coordination polymer or whether regions of the lamellae break down into Cu clusters that rapidly grow with a lateral preference. For example, amorphous intermediates might be involved, as recently discovered for the macromolecular template-directed carbonate crystallization and other metal NCs.^81,83−85^

## Conclusions

In conclusion, we have developed and studied the synthesis of Cu NCs via Cu(I) phosphonate lamella intermediates by using different lengths of aliphatic phosphonic acid ligands. We have discovered that the chain length of the ligands controls the structure of the lamellae and, thus, their thermal stability along with the reduction kinetics of Cu(I) to Cu(0). With this new knowledge, we have demonstrated that anisotropic Cu NCs, mostly triangular plates, form from the more thermally robust short-chain lamellae, which exhibit the slowest reduction kinetics. On the contrary, spherical Cu NCs are the reaction product of the long-chain lamellae.

This study highlights that the rational selection of organic ligands during colloidal NC synthesis should take all of their roles in the reaction into account, which goes beyond their most trivial function as surface passivating agents.

We envision that the future development of using lamellae as well-defined prenucleation intermediates will enable greater control in the synthesis of Cu and of other non-noble metal NCs. For example, further tuning of the reduction kinetics can provide access to other shapes, including nanosheets with tunable thicknesses. Being able to tune the elemental composition by combining lamellae of different transition metals will greatly advance their use in catalytic and energy applications.^32−40^

## Supporting Information Available

Experimental methods, additional TEM images and size analyses, and spectroscopic data (FT-IR, XPS, XAS, and XRD) (PDF)

## Notes

The data underlying this study are openly available in Zenodo at https://doi.org/10.5281/zenodo.6362954.
