# Supplementary material for: Copper Phosphonate Lamella Intermediates Control the Shape of Colloidal Copper Nanocrystals
Source: J Am Chem Soc. 2022 Jun 30;144(27):12261–71. doi: 10.1021/jacs.2c03489 (PMC9284559; doi:10.1021/jacs.2c03489)
Supplement: Supplementary file 1 — ja2c03489_si_001.pdf [file ja2c03489_si_001.pdf]

Supporting Information

Copper-Phosphonate Lamella Intermediates Control the Shape of Colloidal Copper Nanocrystals

James R. Pankhurst[a], Laia Castilla-Amorós[a], Dragos C. Stoian[b], Jan Vavra[a], Valeria Mantella[a], Petru P. Albertini[a], Raffaella Buonsanti[a]*

[a] Laboratory of Nanochemistry for Energy (LNCE), Institute of Chemical Sciences and Engineering (ISIC), École Polytechnique Fédérale de Lausanne (EPFL), Rue de l'Industrie 17, 1950 Sion, Switzerland

[b] The Swiss-Norwegian Beamlines, European Synchrotron Radiation Facility (ESRF), 38000 Grenoble, France

* Corresponding author: raffaella.buonsanti@epfl.ch

Experimental Section

Chemicals

Copper(I) acetate (Cu(OAc), 97%,), *n*-tetradecylphosphonic acid (TDPA, 98%), *n*-decylphosphonic acid (DPA, 97%), tri-*n*-octylamine (TOA, 98%), oleylamine (OLAM, 70%), octadecylamine (ODAM, 99%), decylamine (DAM, 95%), hexylamine (HAM, 99%) and LaB_6 (NIST standard) were purchased from Sigma-Aldrich. *n*-Octylphosphonic acid (OPA, 99%), *n*-butylphosphonic acid (BPA, 98%) and toluene (anhydrous, 99.8%) were purchased from ABCR GmbH. *n*-Hexylphosphonic acid (HPA, 98%) was purchased from TCI Europe. Octadec-1-ene (ODE, 90%), hexane (anhydrous, 99%) and ethanol (anhydrous, 95%) were purchased from Chemie Brunschwig AG. Acetone (anhydrous, 99.8%) was purchased from Acros. All reagents were used as purchased without any additional purification.

General synthetic procedures

All reagents and anhydrous solvents were stored under a dry N_2 atmosphere. All manipulations in the synthesis of Cu NCs were also made under a dry N_2 atmosphere using Schlenk-line techniques and a glove box. All NC products were washed, stored and analyzed using anhydrous solvents. All volumes were dispensed using Eppendorf microliter pipettes. All glassware was thoroughly cleaned and dried overnight in an oven at 150 °C before each reaction. Concentrated nitric acid was used to remove any metallic residues from the reaction flask after each reaction and the flask was washed thoroughly with milli-Q water.

In a typical reaction, a three-neck round-bottom flask was equipped with a reflux condenser that was attached to a N_2 supply. The flask was also equipped with a thermocouple connected to a temperature control box. The third neck was sealed with a rubber septum, to be removed for adding reagents and solvents. The glassware was assembled while hot and was then purged under dynamic vacuum three times, refilling with N_2 after each cycle. The TOA solvent was added to the flask (where used, OLAM was also added in this step) and the solvent was then degassed with stirring under dynamic vacuum while heating at 130 °C for 45 minutes. Heating was done using an electrothermal heating mantle and was regulated by the temperature control box; the flask was insulated with glass wool during heating. After cooling to approximately 50 °C, Cu(OAc) and the phosphonic acid ligand were added under a flow of N_2, forming a green suspension. While the specific reaction temperatures were varied, the reaction mixtures were generally heated to one temperature (T_1, between 130 and 180 °C) in order to build up the lamella intermediate and then heated to a second temperature (T_2, between 180 and 270 °C) in order to reduce the intermediate to metallic Cu NCs. At the end of the reaction, the mixture was cooled to 100 °C using a compressed air gun, after removing the heating mantle and glass wool insulation. At 90 °C, the mixture was removed from the flask using a needle and syringe and the mixture was transferred to N_2-filled glass vials fitted with rubber septa. In a glove box, the reaction mixture was divided into two centrifugation tubes (approximately 5.5 mL of reaction mixture volume in each tube) and each portion was mixed with hexane (6 mL) and ethanol (6 mL) for washing. The NC products were collected by centrifugation (at 13,000 rpm for small, spherical products and at 6000 rpm for large, spherical or 2D products) for 10 minutes. This washing and centrifugation process was repeated after resuspending the NCs; the process was repeated only once when isolating lamella intermediates (to avoid damage to the assemblies by excessive ligand stripping) and was repeated twice when isolating metallic NCs. The NC products were finally recovered and stored in 6000 μL of toluene.

Specific synthetic procedures

Spherical Cu NCs of different sizes are reported in **Figure 3**. They were obtained according to the following specific procedures.

Synthesis of 6 nm spheres

TOA (10 mL) was degassed in a round-bottom flask before adding Cu(OAc) (123 mg, 1 mmol) and TDPA (139 mg, 0.5 mmol), forming a green suspension. The mixture was heated to 180 ºC, during which time the mixture turned yellow and then formed a cloudy tan suspension. After 30 minutes, the mixture was heated to 270 ºC, quickly turning red and then opaque red/brown around 200–210 ºC. After 30 minutes, the mixture was cooled, collected and purified. Particle diameter: 6.4 ± 0.3 nm.

Synthesis of 7 nm spheres

1) TOA (10 mL) was degassed in a round-bottom flask before adding Cu(OAc) (123 mg, 1 mmol) and TDPA (139 mg, 0.5 mmol), forming a green suspension. The mixture was heated to 180 ºC, during which time the mixture turned yellow and then formed a cloudy tan suspension. After 30 minutes, the mixture was heated to 250 ºC, quickly turning red and then opaque red/brown around 200–210 ºC. After 30 minutes, the mixture was cooled, collected and purified. Particle diameter: 7.0 ± 0.7 nm.

2) TOA (10 mL) was degassed in a round-bottom flask before adding Cu(OAc) (123 mg, 1 mmol) and DPA (111 mg, 0.5 mmol), forming a green suspension. The mixture was heated to 180 ºC, during which time the mixture turned yellow and then formed a cloudy tan suspension. After 30 minutes, the mixture was heated to 270 ºC, quickly turning red and then opaque red/brown around 200–210 ºC. After 30 minutes, the mixture was cooled, collected and purified. Particle diameter: 7.0 ± 0.8 nm.

Synthesis of 8 nm spheres

1) TOA (10 mL) was degassed in a round-bottom flask before adding Cu(OAc) (123 mg, 1 mmol) and TDPA (139 mg, 0.5 mmol), forming a green suspension. The mixture was heated to 180 ºC, during which time the mixture turned yellow and then formed a cloudy tan suspension. After 30 minutes, the mixture was heated to 230 ºC, quickly turning red and then opaque red/brown around 200–210 ºC. After 30 minutes, the mixture was cooled, collected and purified. Particle diameter: 8.5 ± 0.5 nm.

2) TOA (10 mL) was degassed in a round-bottom flask before adding Cu(OAc) (123 mg, 1 mmol) and DPA (111 mg, 0.5 mmol), forming a green suspension. The mixture was heated to 180 ºC, during which time the mixture turned yellow and then formed a cloudy tan suspension. After 30 minutes, the mixture was heated to 250 ºC, quickly turning red and then opaque red/brown around 200–210 ºC. After 30 minutes, the mixture was cooled, collected and purified. Particle diameter: 8.0 ± 1.1 nm.

Synthesis of 11 nm spheres

1) TOA (10 mL) was degassed in a round-bottom flask before adding Cu(OAc) (123 mg, 1 mmol) and TDPA (139 mg, 0.5 mmol), forming a green suspension. The mixture was heated to 180 ºC, during which time the mixture turned yellow and then formed a cloudy tan suspension. After 30 minutes, the mixture was heated to 210 ºC, quickly turning red and then opaque red/brown around 200–210 ºC. After 30 minutes, the mixture was cooled, collected and purified. Particle diameter: 11.3 ± 1.0 nm.

2) TOA (10 mL) was degassed in a round-bottom flask before adding Cu(OAc) (123 mg, 1 mmol) and DPA (111 mg, 0.5 mmol), forming a green suspension. The mixture was heated to 180 ºC, during which time the mixture turned yellow and then formed a cloudy tan suspension. After 30 minutes, the mixture was heated to 230 ºC, quickly turning red and then opaque red/brown around 200–210 ºC. After 30 minutes, the mixture was cooled, collected and purified. Particle diameter: 11.0 ± 1.2 nm.

Synthesis of 13 nm spheres

TOA (10 mL) was degassed in a round-bottom flask before adding Cu(OAc) (123 mg, 1 mmol) and DPA (111 mg, 0.5 mmol), forming a green suspension. The mixture was heated to 180 ºC, during which time the mixture turned yellow and then formed a cloudy tan suspension. After 30 minutes, the mixture was heated to 210 ºC, quickly turning red and then opaque red/brown around 200–210 ºC. After 30 minutes, the mixture was cooled, collected and purified. Particle diameter: 13.0 ± 2.0 nm.

Synthesis of 28 nm spheres

TOA (10 mL) was degassed in a round-bottom flask before adding Cu(OAc) (123 mg, 1 mmol) and HPA (111 mg, 0.5 mmol), forming a green suspension. The mixture was heated to 180 ºC, during which time the mixture turned yellow and then formed a cloudy tan suspension. After

30 minutes, the mixture was heated to 250 ºC, slowly turning red and then opaque red/brown around 220 ºC. After 30 minutes, the mixture was cooled, collected and purified. Particle diameter: 27.6 ± 7.5 nm.

Synthesis of 52 nm spheres

TOA (10 mL) was degassed in a round-bottom flask before adding Cu(OAc) (123 mg, 1 mmol) and BPA (69 mg, 0.5 mmol), forming a green suspension. The mixture was heated to 180 ºC, during which time the mixture turned yellow and then formed a cloudy tan suspension. After 30 minutes, the mixture was heated to 270 ºC, slowly turning red and then opaque red/brown around 230–240 ºC. After 30 minutes, the mixture was cooled, collected and purified. Particle diameter: 52.4 ± 11.0 nm.

Synthesis of circular lamella sheets

TOA (9.67 mL) and OLAM (0.32 mL, 1 mmol) were degassed in a round-bottom flask before adding Cu(OAc) (123 mg, 1 mmol) and BPA (69 mg, 0.5 mmol), forming a green suspension. The mixture was heated to 150 ºC, during which time the mixture turned yellow and then formed a cloudy tan suspension. After 30 minutes, the mixture appeared light brick-red. The mixture was cooled, collected and purified.

Synthesis of 400 nm triangles

TOA (9.67 mL) and OLAM (0.32 mL, 1 mmol) were degassed in a round-bottom flask before adding Cu(OAc) (123 mg, 1 mmol) and BPA (69 mg, 0.5 mmol), forming a green suspension. The mixture was heated to 150 ºC, during which time the mixture turned yellow and then formed a cloudy tan suspension. After 30 minutes, the mixture appeared light brick-red. The mixture was heated to 220 ºC, slowly turning red and then opaque red/brown. After 30 minutes, the mixture was cooled, collected and purified. For TEM imaging, some residual lamella was removed with an additional washing step using 4-*tert*-butyl-pyridine (20 μL per 200 μL NC suspension in toluene) to break up the coordination polymer. After centrifugation, the NCs were washed with 1:1 hexane / ethanol to remove any pyridine. The final NCs were resuspended in the same initial volume of toluene. Particle edge length: 385 ± 179 nm.

Ex-situ characterization

Transmission electron microscopy (TEM)

Samples were prepared by drop-casting toluene NC suspensions onto carbon-coated copper TEM grids (Ted Pella, Inc.). Before drop-casting, NC suspensions were diluted with an equal volume of toluene and sonicated. Low-resolution TEM and selected-area electron diffraction (SAED) images were measured using a Thermo Fisher Tecnai-Spirit microscope operating at 120 kV. High-resolution TEM and high-angle annular dark-field STEM images were recorded using a double-corrected Thermo Fisher Titan microscope operating at 300 kV. Size distribution analysis was carried out using ImageJ software, where at least 200 particles were measured.

X-ray diffraction (XRD)

Samples were measured on 2.5 × 2.5 cm^2 silicon substrates that were cleaned by sonication in acetone and then hexane. Samples were prepared by drop-casting toluene NC suspensions on the substrate and leaving the toluene to evaporate. Serial drops were cast in order to accumulate adequate amounts of NCs for analysis. A Bruker D8 Advance diffractometer was used, which is equipped with a Lynxeye one-dimensional detector and operates at 40 kV and 40 mA, using a Cu K_α source with a wavelength of 1.54 Å. Samples were rotated during the measurement, which was made between 2θ values of 2 and 65 degrees.

Inductively-coupled plasma optical-emission spectroscopy (ICP-OES)

Measurements were made using an Agilent 5100 model. Calibration curves were made after diluting a Cu standard solution to eight different concentrations, spanning 1 ppm to 50 ppm. Sample solutions were made by evaporating the organic solvent from an aliquot of a Cu NC suspension and digesting the NCs in 70% high-purity HNO_3; milli-Q water was then added to dilute the HNO_3 to 2% for analysis.

Fourier-transform infrared spectroscopy (FT-IR)

Background scans were obtained for the clean ATR plate in air. Samples were prepared by sonicating toluene NC suspensions and then drop-casting directly onto the ATR plate, leaving the sample to air-dry. Spectra were recorded using a total of 16 scans with a resolution of 4 cm^{-1}.

X-ray photoelectron spectroscopy (XPS)

Samples were measured on 1 × 1 cm^2 silicon substrates that were cleaned by sonication in acetone and then hexane. Samples were prepared by drop-casting toluene NC suspensions onto the substrate and evaporating to dryness on a flat sample stage. Serial drops were cast in order to build up adequate amounts of NCs for analysis. Measurements were made using an Axis Supra instrument (Kratos Analytical), using the monochromated K_α X-ray line of an Al anode. A pass energy of 20 eV was used with a step size of 0.2 eV. All samples were electrically isolated from the substrate and charges were compensated. All spectra were referenced using the C–C peak in the C 1*s* region, which was set to 284.8 eV.

***In-situ* reaction monitoring**

Reaction monitoring using *in-situ* X-ray absorption spectroscopy (XAS) and X-ray diffraction (XRD) techniques was carried out on the Swiss-Norwegian beamline (SNBL, BM31) at the European Synchrotron Radiation Facility (ESRF) in Grenoble, France, using an operating current of 200 mA.

XAS spectra were acquired at the Cu K-edge (8.9789 keV) using a Si(111) double crystal monochromator (DCM) in transmission mode (using 30 cm ion chambers as detectors) using a continuous scan between 8940 and 9060 eV, a step-size of 0.75 eV and 100 ms integration time per data point. The total scan duration was *ca.* 18 s. A Cu foil was used for energy calibration and the beam cross section was *ca.* 4 × 0.5 mm^2 (width × height). XRD data were collected with a 2D DEXELA 2923 plate detector using a Si(111) DCM at a wavelength of 0.336876 Å (corresponding to an energy of 36.8 keV), using a focused beam with a cross section of 30 × 100 μm^2 (width × height). Averaging protocols and azimuthal integration were performed with the pyFAI software using lanthanum hexaboride (LaB_6) fine powder as a standard[1]; the XRD patterns were averaged over a period of 10 s. The sample-to-detector distance was adjusted and optimized for the best signal at low 2θ values. *D*-spacing values were obtained by solving Bragg's equation $n\lambda = 2d\sin\theta$, for $n = 1$.

The reaction flasks for these experiments mimic a typical three-neck round-bottom flask and can be fitted with a reflux condenser (connected to a N_2 supply) and a thermocouple, leaving one neck for adding and removing reagents and solvents. The same flasks have been used by us in previous *in-situ* synchrotron studies.[2,3] One side of the flask is fitted with a Kapton window (12.5 µm thick, HN) sealed with a Kalrez O-ring and PTFE tightening ring, which

seals the reaction volume and is leak-free even in high-temperature (250 °C) amine solvents. The flasks are also equipped with glass path guides that can be adjusted to fine-tune the depth of the X-ray flight path in the reaction volume; this path guide is also fitted with an O-ring-sealed Kapton window. The use of Kapton windows allows experiments to be carried out with soft X-rays in the 5 keV range. The diameter of the outside Kapton window is 22 mm and that of the inside window is 6 mm. The flight path was 10 mm for XAS experiments and 2 mm for XRD experiments. Heating of the flask is achieved using thermoelectric heating cords that are regulated remotely in the beamline control room using a temperature control box. Adequate stirring of the mixture was achieved using a thin-profile magnetic stir plate, which was crucial in order to obtain good data quality, especially in diffraction experiments.

The *in-situ* experiments were carried out similarly to the syntheses carried out in the lab, with some modification as the *in-situ* cell cannot be placed under vacuum. Solvent mixtures were previously degassed in pre-measured portions and solid reagents were pre-weighed. After purging the flask with a flow of N_2 for some minutes, the solids were added to the flask, followed by the solvent. This procedure resulted in yellow / green suspensions and indicates that no oxidation of the copper precursor takes place (which would form a blue suspension).

Alignment of the reaction flask with the X-ray beam was done using a precise sample stage that can be rotated and translated in all x,y,z directions.

X-ray absorption spectroscopy, principal component analysis (PCA) and multivariate curve resolution (MCR-ALS).

The PrestoPronto package (for handling large datasets)[4], Athena and Artemis (in the Demeter package)[5] were used for XAS data processing (extraction, background subtraction, normalization, *etc*.). 3 scans were averaged for all *in-situ* XAS experiments in order to improve the signal-to-noise ratio. A multivariate curve resolution with alternating least square (MCR-ALS) mathematical algorithm[6–8] was employed for the treatment of the large datasets generated during the Cu NC synthesis.

MCR-ALS is a very popular data-reduction method used to extract kinetically pure spectral components and their corresponding concentration behaviors from an unknown mixture. This is done in a blind manner and no references are needed *a priori*. The MCR-ALS model is described by the equation $\mathbf{D} = \mathbf{C}\mathbf{S}^t + \mathbf{E}$, where $\mathbf{D}$ is the initial matrix composed of all the

spectroscopic data, **C** represents the extracted concentration profiles after minimization, **S** corresponds to the extracted pure spectra profiles (**t** is the transposed matrix), and **E** is the error matrix containing the residuals and a close approximation of the experimental error of the measurement. The MCR-ALS graphical user interface for MATLAB®[9] was used to process the Cu K-edge time-resolved XAS datasets by employing positive constraints for both spectra and concentration profiles, and closure constraints for the concentration profiles (since the overall Cu concentration was constant throughout the reaction).

Principal component analysis (PrestoPronto package)[4] served in performing the rank analysis and deciding on the number of components to be employed during the MCR-ALS run. Statistically, there was a clear trend supporting the existence of a single reaction intermediate for all of the reactions studied *i.e.,* the transition from Cu(I) to Cu(0) occurs *via* an intermediate species. The introduction of a 4th component to the model was statistically insignificant in all cases.

Molecular mechanics models of the lamella intermediates

Very simple molecular mechanics models of the lamella structure were made using Avogadro software. Structures were drawn using two phosphonate molecules connected by a single Cu atom. A near-linear coordination geometry was used in the guess structure as this geometry is implied by the *in-situ* X-ray absorption data. Geometries were optimized using the UFF force field in Avogadro. The length of the Cu-phosphonate unit was then taken as the distance from a phosphorus atom to the terminal hydrogen atom on the connecting alkyl chain. The lamella spacing was predicted by doubling this distance. Note that for graphical representation of the lamella, as in **Figure 2B**, two identical models were imported and arranged in Blender ‘by eye’; the models shown in the Figure were not calculated directly as a ‘bimolecular’ system.

Supplementary figures and tables to the main text

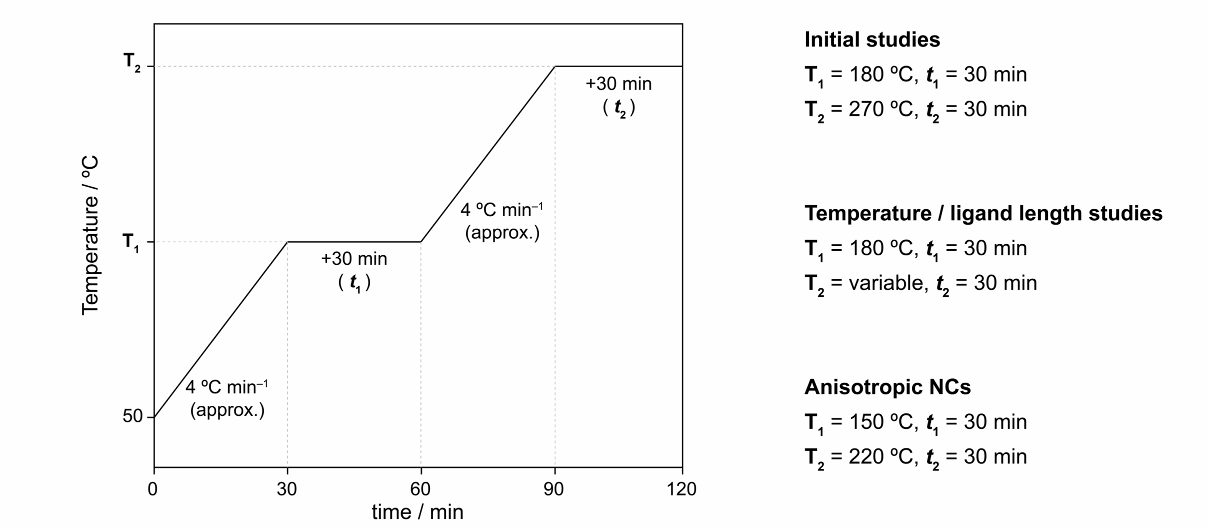

Figure S1. Heating profiles for the syntheses of Cu NCs described in the main text. The different T_1 and T_2 values for different studies are specified.

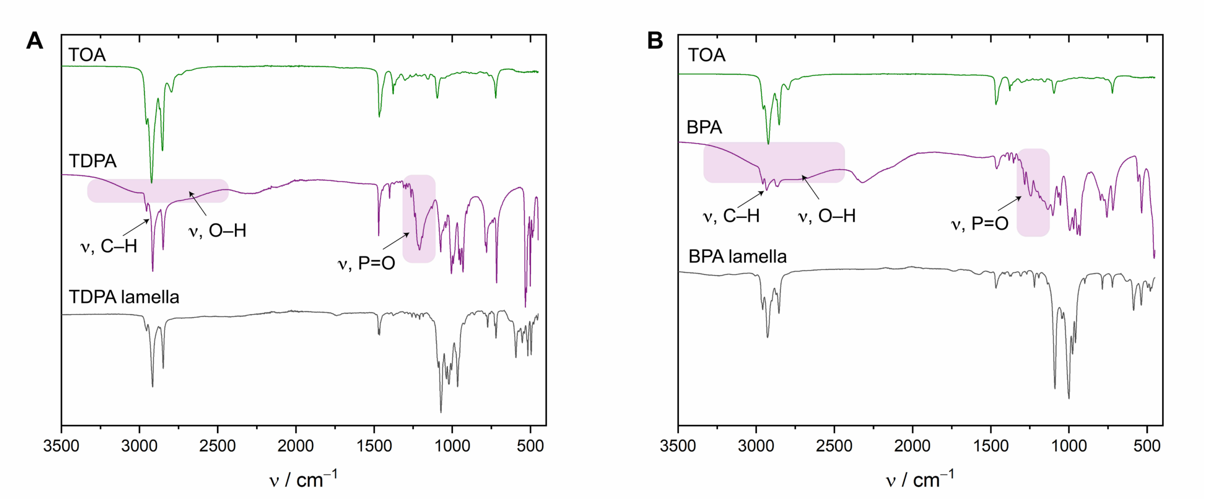

Figure S2. FT-IR spectra for (**A**) the long-chain TDPA lamella and (**B**) the short-chain BPA lamella. Reference spectra for the TOA reaction solvent and respective phosphonic acid ligands are also shown for comparison. For the phosphonic acid spectra, the ν, P=O and ν, O–H absorption bands are highlighted. These peaks are not observed in the lamellae spectra, indicating deprotonation of the phosphonic acid groups and coordination to Cu(I) through all three available oxygen atoms in the -PO_3^{2-} group.

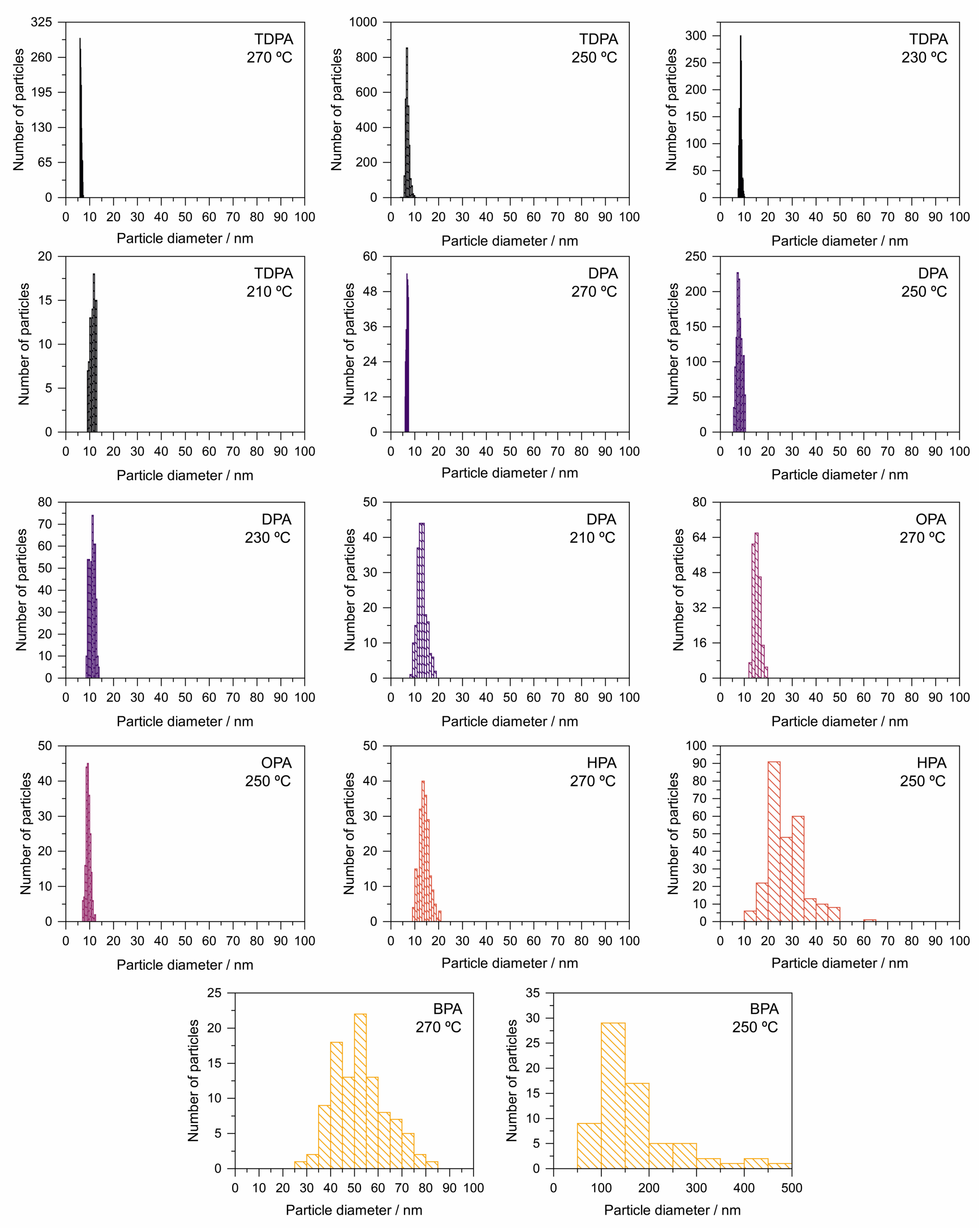

Figure S3. Size analysis of spherical Cu NC products from reactions with different phosphonic acid ligands at different temperatures. The ligand used and final reaction temperature are shown inset on each graph. Clearly, spherical NC products formed from short-chain lamellae suffer from poor size dispersity.

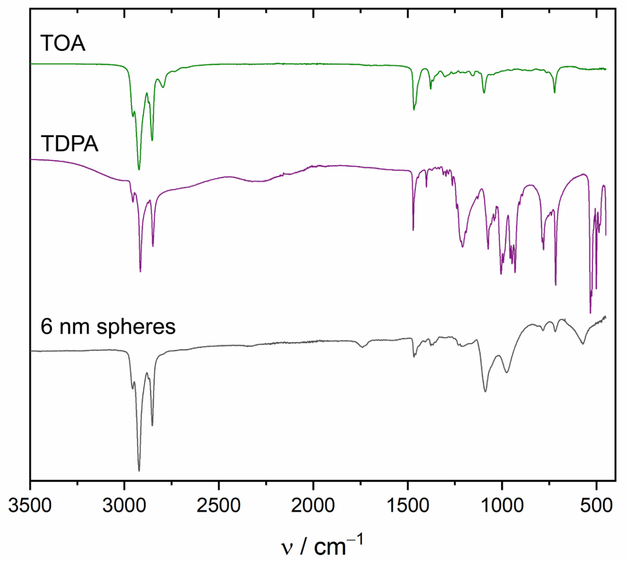

Figure S4. FT-IR spectra for 6 nm, spherical Cu NCs synthesized from TDPA.

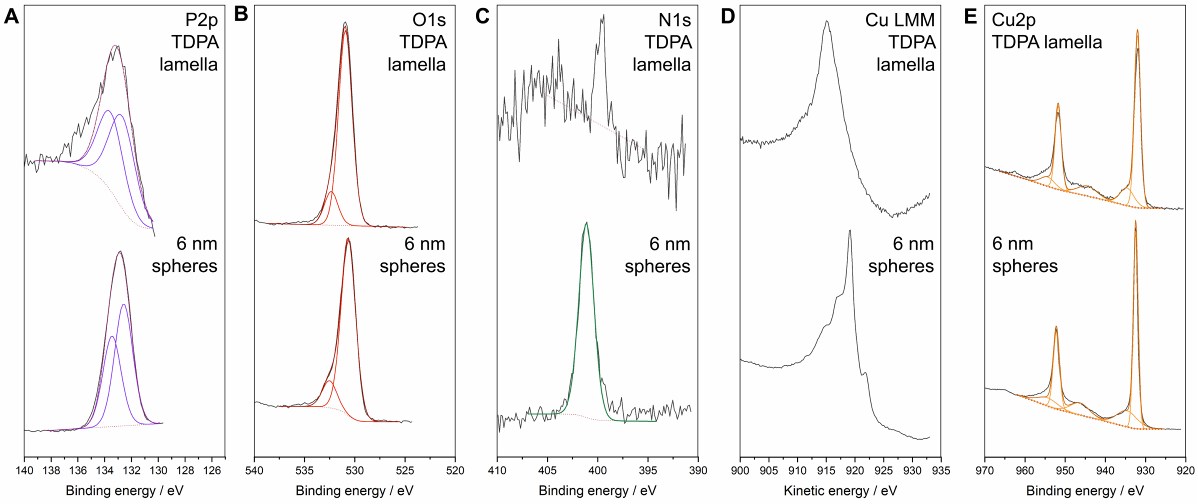

Figure S5. XPS spectra for (**A**) P2*p* (**B**) O1*s* (**C**) N1*s* (**D**) Cu LMM and (**E**) Cu2*p* regions, for the isolated TDPA lamella intermediate and the resulting 6 nm, spherical Cu NC product.

The TEM images in **Figure 3** along with the size distribution analysis in **Figure S3** show that longer ligands and higher temperatures generally favor smaller, more monodisperse NCs. The larger NC sizes with shorter ligands are attributed to the reduced sterics and faster binding dynamics of small molecules.[10–13] The FT-IR analysis in **Figure S4** and XPS analysis in **Figure S5** indicate that both TOA and the phosphonate ligands are present on the surface of the spherical NCs. Furthermore, in the XPS the Cu2*p* and Cu LMM spectra indicate that Cu is in the +1 oxidation state in the lamella while it is metallic in the spherical NCs.

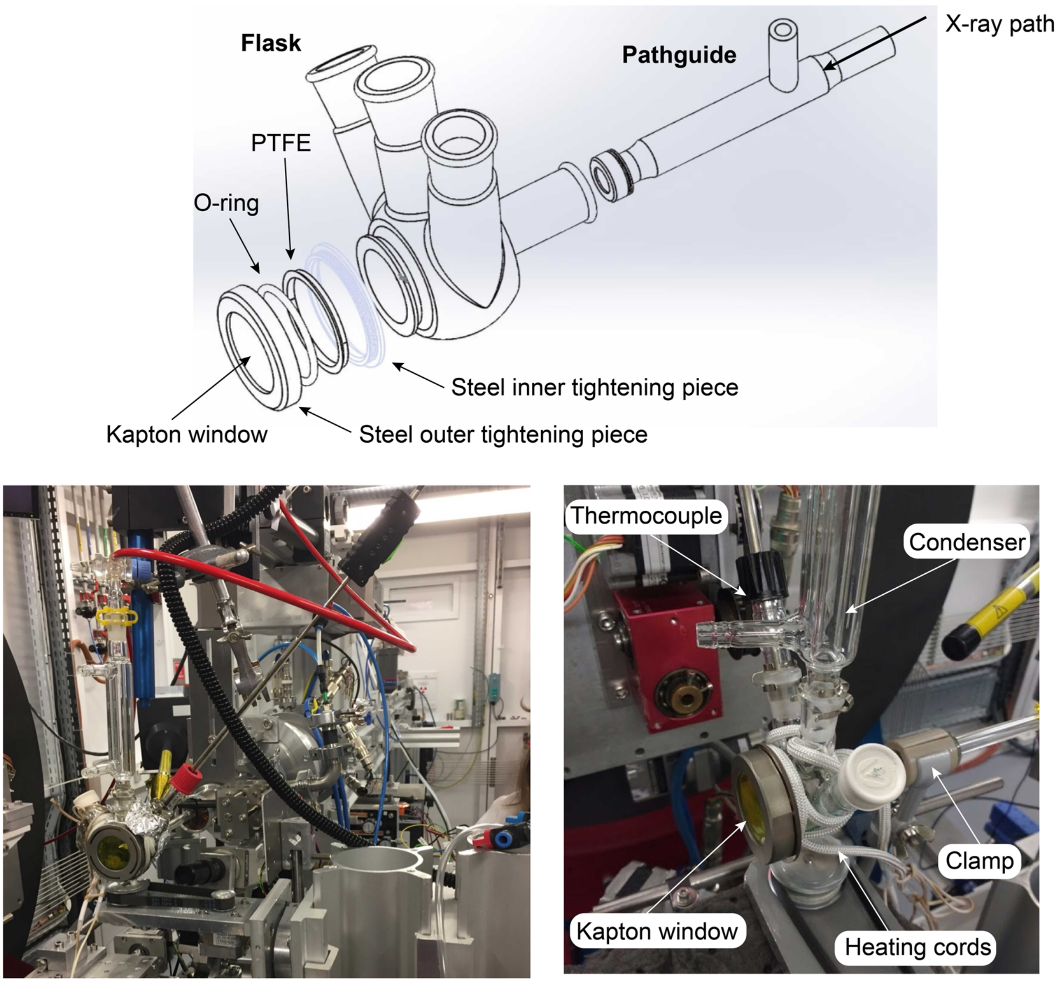

Figure S6. **Top**: schematic of the *in-situ* flask, showing the flask and pathguide components; the Kapton window assembly at the front of the flask is shown in detail and an equivalent assembly is also used on the pathguide piece. **Bottom**: photographs of the *in-situ* flask installed on the beamline at the ESRF (BM-31).

A note regarding in-situ XAS data

For the following *in-situ* XAS spectra, essentially identical observations were made for all reactions, regardless of the phosphonic acid ligand used. This finding shows that the different lamellae do not impact the Cu(I) reduction temperature and that metallic Cu is formed at a similar temperature at a similar rate. However, XAS does not reveal the form of the metallic Cu(0) and cannot differentiate between Cu nuclei dispersed in solution or trapped within a lamella intermediate.

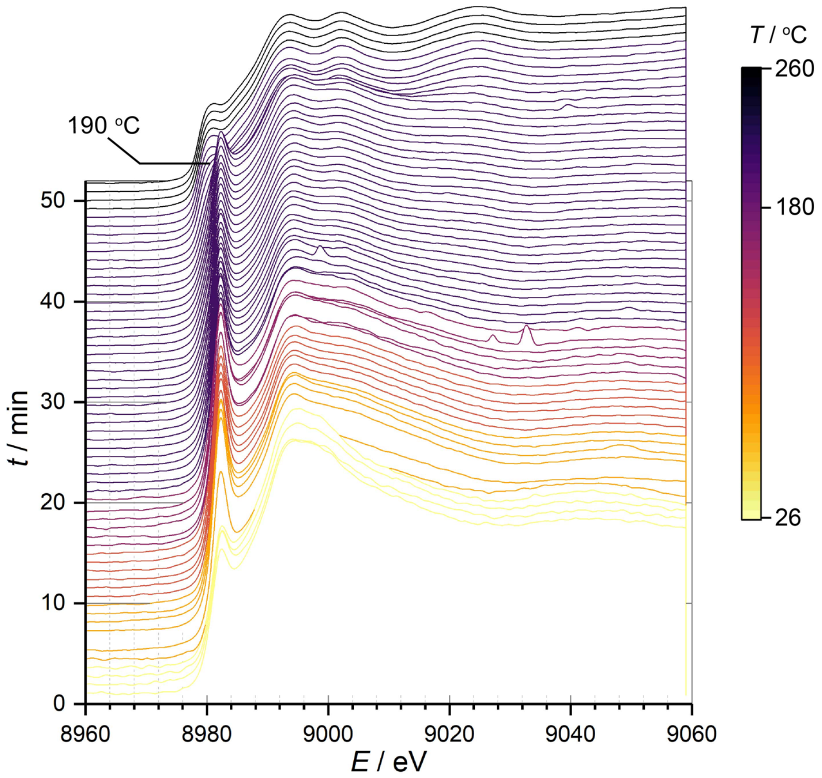

Figure S7. *In-situ* Cu K-edge XAS spectra plotted against time with the reaction temperature indicated by color. The reaction contained Cu(OAc) (1.6 mmol), TDPA (0.8 mmol) and TOA (16 mL) as the solvent.

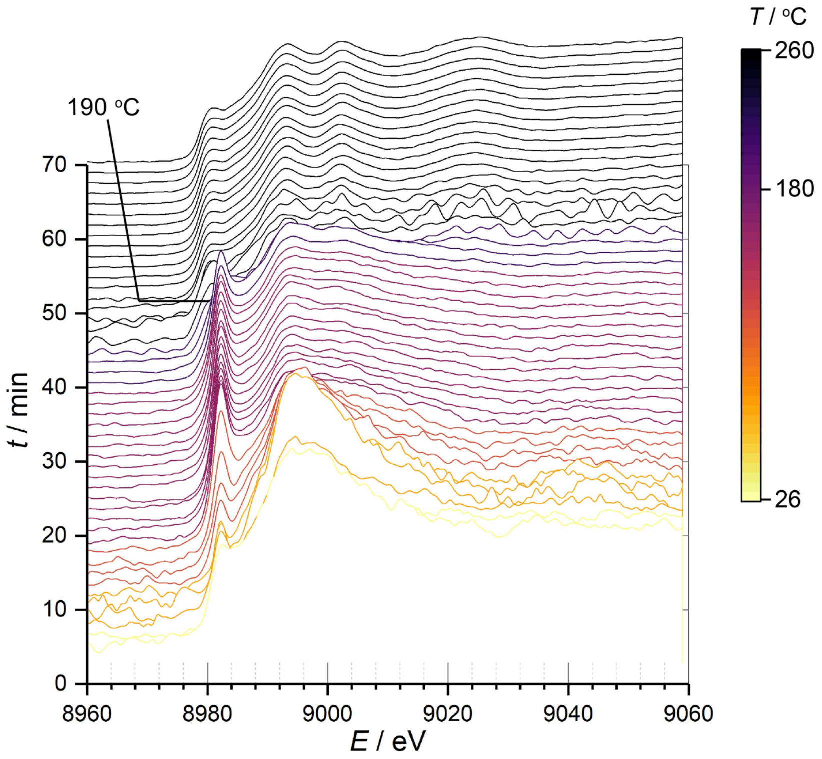

Figure S8. *In-situ* Cu K-edge XAS spectra plotted against time with the reaction temperature indicated by color. The reaction contained Cu(OAc) (1.6 mmol), OPA (0.8 mmol) and TOA (16 mL) as the solvent.

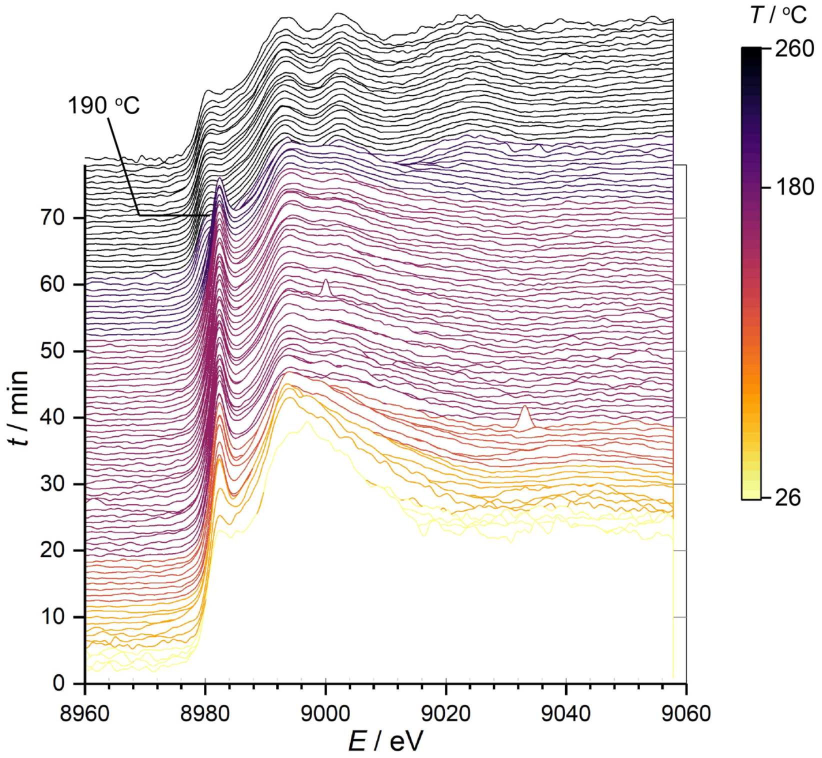

Figure S9. *In-situ* Cu K-edge XAS spectra plotted against time with the reaction temperature indicated by color. The reaction contained Cu(OAc) (1.6 mmol), HPA (0.8 mmol) and TOA (16 mL) as the solvent.

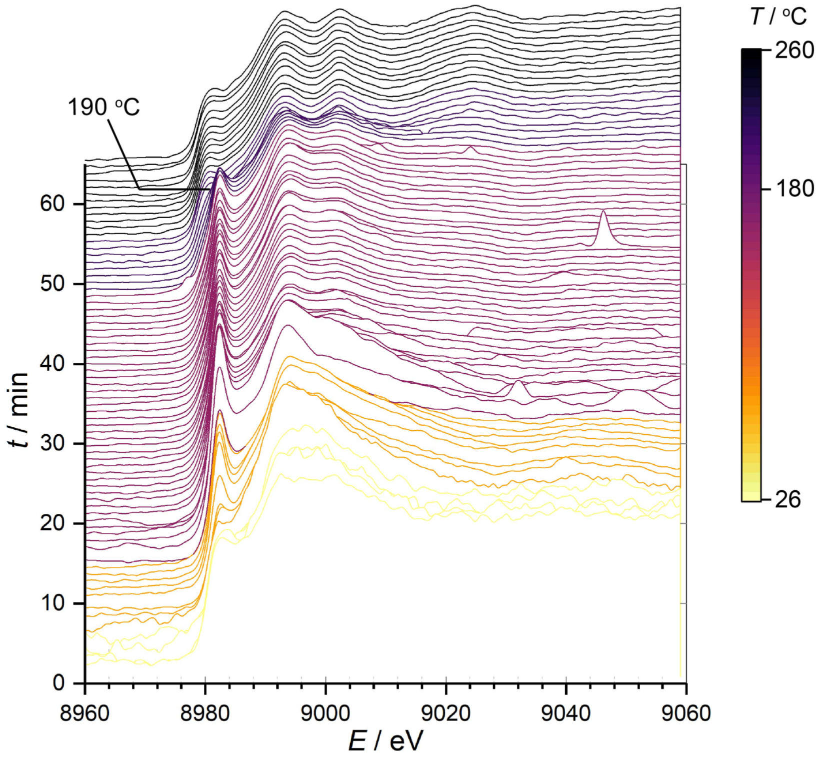

Figure S10. *In-situ* Cu K-edge XAS spectra plotted against time with the reaction temperature indicated by color. The reaction contained Cu(OAc) (1.6 mmol), BPA (0.8 mmol) and TOA (16 mL) as the solvent.

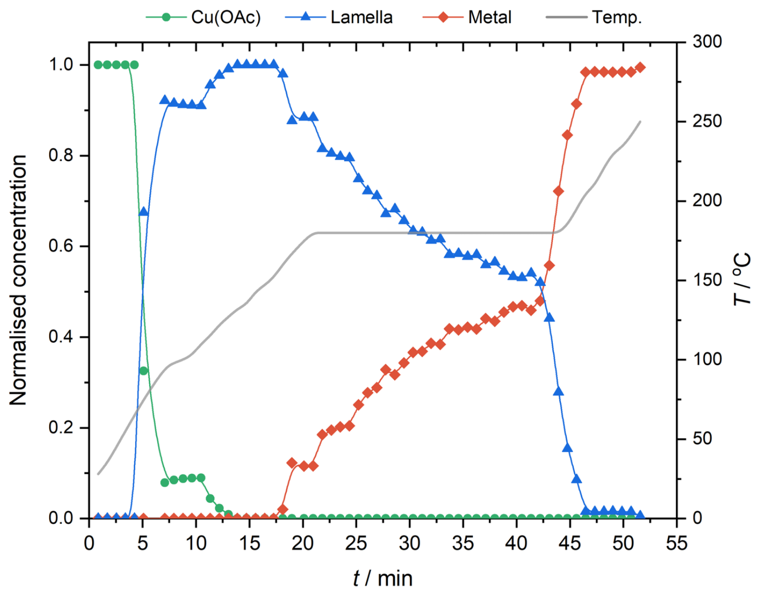

Figure S11. Concentration profiles for the Cu(OAc), Cu(I) lamella and metallic Cu components from the *in-situ* Cu K-edge XAS study of the reaction with TDPA. Concentrations are normalized and are derived from principal component analysis. The reaction temperature is also shown.

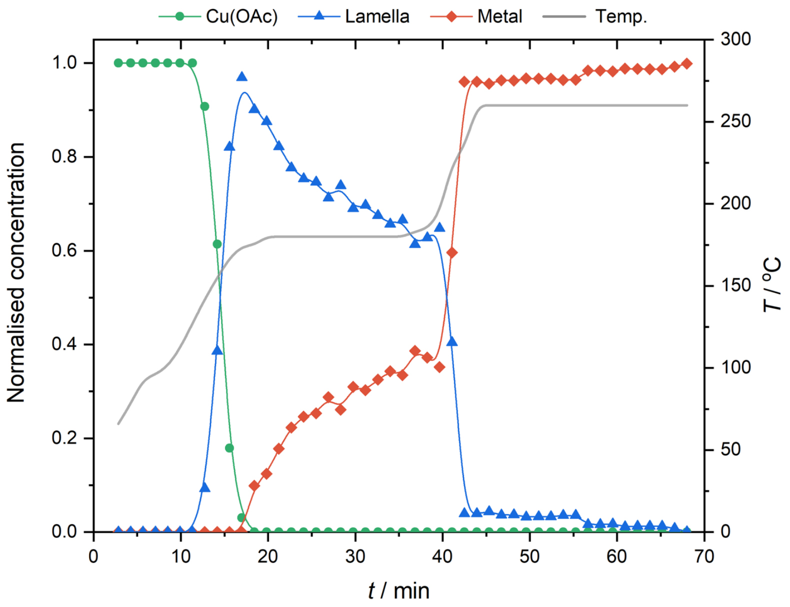

Figure S12. Concentration profiles for the Cu(OAc), Cu(I) lamella and metallic Cu components from the *in-situ* Cu K-edge XAS study of the reaction with OPA. Concentrations are normalized and are derived from principal component analysis. The reaction temperature is also shown.

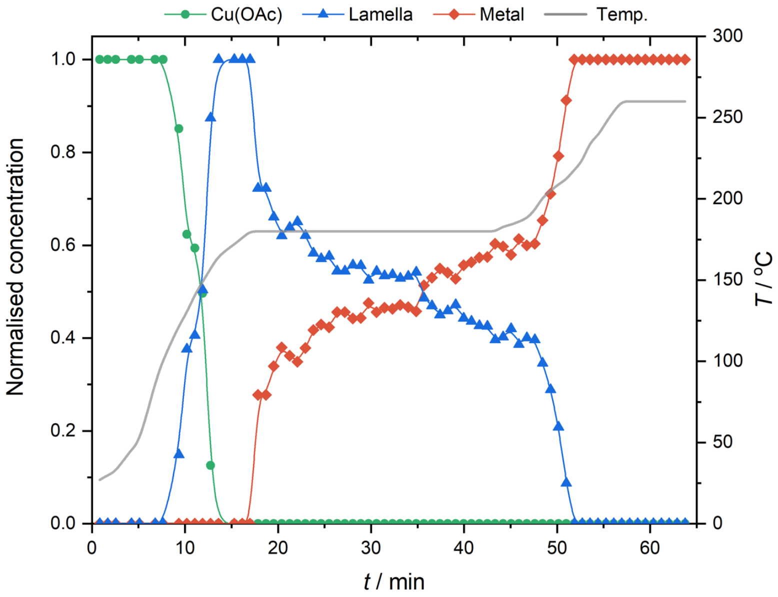

Figure S13. Concentration profiles for the Cu(OAc), Cu(I) lamella and metallic Cu components from the *in-situ* Cu K-edge XAS study of the reaction with HPA. Concentrations are normalized and are derived from principal component analysis. The reaction temperature is also shown.

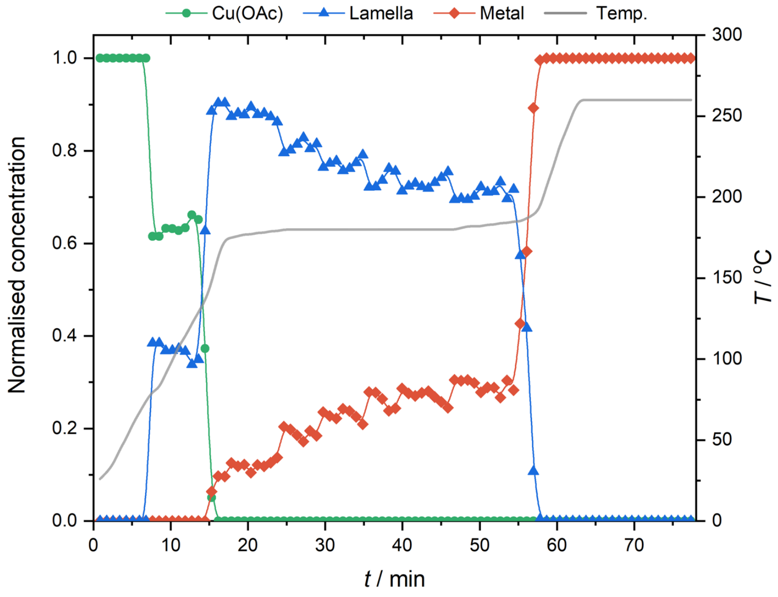

Figure S14. Concentration profiles for the Cu(OAc), Cu(I) lamella and metallic Cu components from the *in-situ* Cu K-edge XAS study of the reaction with BPA. Concentrations are normalized and are derived from principal component analysis. The reaction temperature is also shown.

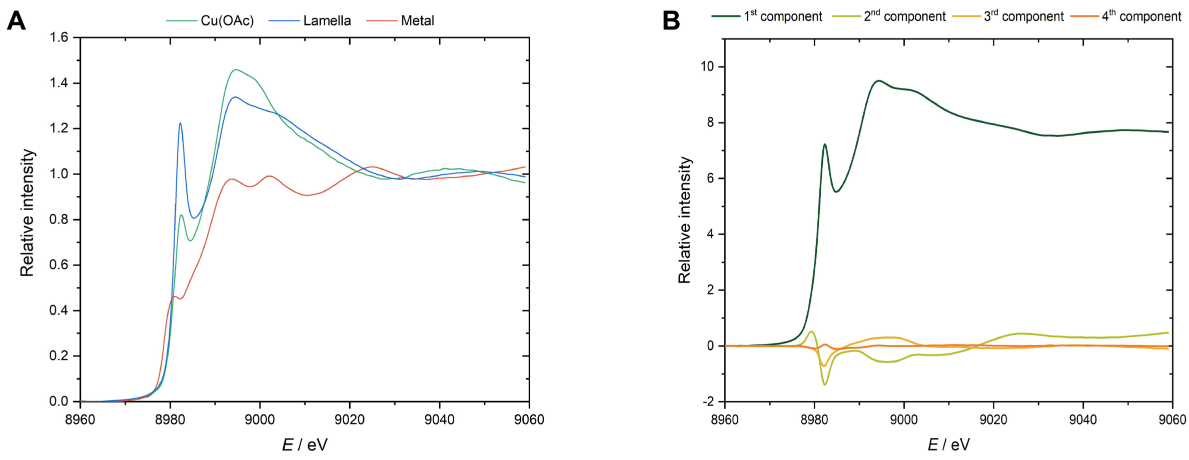

Figure S15. Principal component analysis of *in-situ* Cu K-edge XAS spectra for the TDPA reaction. **A**) Extracted spectra for the Cu(OAc), Cu(I) lamella and metallic Cu components. **B**) Eigenvalue analysis, showing that only the first 3 components make a significant contribution to the deconvolution; the eigenvalues for the first 5 components are 6770, 22.5, 5.28, 0.203 and 0.1, respectively.

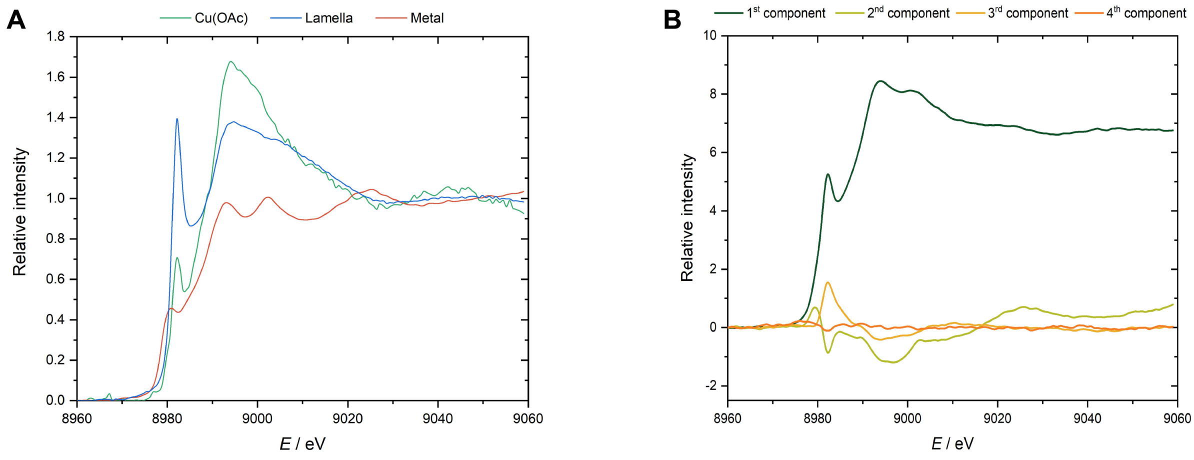

Figure S16. Principal component analysis of *in-situ* Cu K-edge XAS spectra for the OPA reaction. **A**) Extracted spectra for the Cu(OAc), Cu(I) lamella and metallic Cu components. **B**) Eigenvalue analysis, showing that only the first 3 components make a significant contribution to the deconvolution; the eigenvalues for the first 5 components are 5110, 36.5, 11.1, 0.723 and 0.499, respectively.

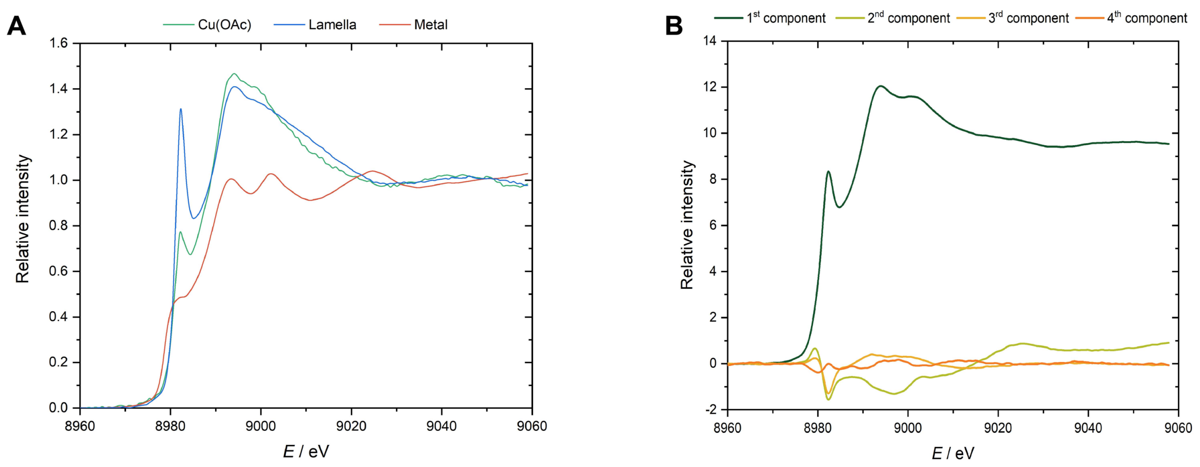

Figure S17. Principal component analysis of *in-situ* Cu K-edge XAS spectra for the HPA reaction. **A**) Extracted spectra for the Cu(OAc), Cu(I) lamella and metallic Cu components. **B**) Eigenvalue analysis, showing that only the first 3 components make a significant contribution to the deconvolution; the eigenvalues for the first 5 components are 7750, 31.2, 6.06, 0.983 and 0.564, respectively.

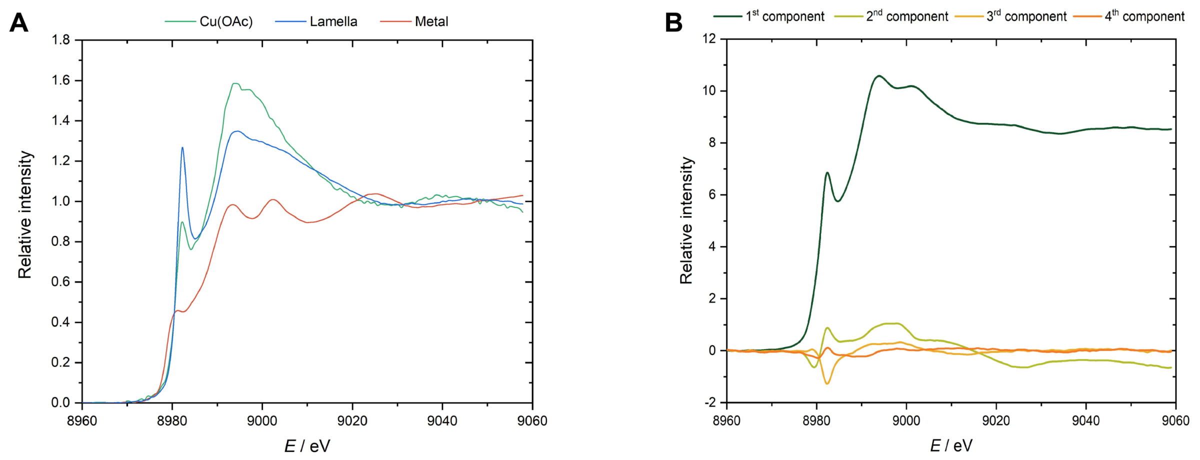

Figure S18. Principal component analysis of *in-situ* Cu K-edge XAS spectra for the BPA reaction. **A**) Extracted spectra for the Cu(OAc), Cu(I) lamella and metallic Cu components. **B**) Eigenvalue analysis, showing that only the first 3 components make a significant contribution to the deconvolution; the eigenvalues for the first 5 components are 10100, 58.8, 7.07, 1.43 and 0.651, respectively.

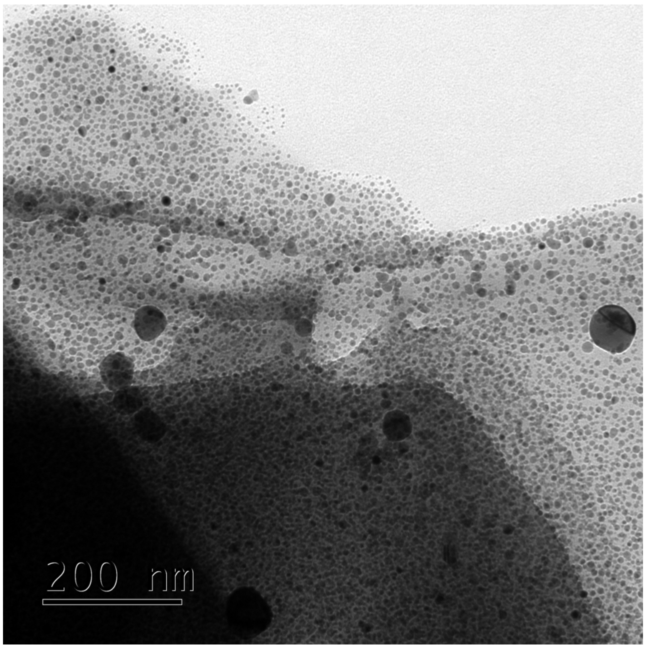

Figure S19. TEM image of the HPA lamella intermediate after stopping the reaction at 180 ºC. The ruffled, low-contrast lamella sheet is covered by, or contains, higher-contrast dots. From the *in-situ* Cu K-edge XAS studies, a mixture of lamella and metallic Cu phases are observed to exist at this temperature. Similar results were obtained for the other phosphonate lamellae.

A note regarding in-situ XRD data

For the following *in-situ* XRD data, the region between $2\theta = 3^o – 6^o$ has been cut as it contains a very large scattering peak from the solvent (confirmed with an XRD measurement where the *in-situ* flask contained TOA only). For all reactions there is a 30-minute temperature plateau at 150 ºC and another at 240 ºC. Some 'doublet' splitting of the peaks is attributed to a geometric effect, where some material was stuck to the Kapton window in the flask, causing diffraction at a different distance to the detector compared to the solution.

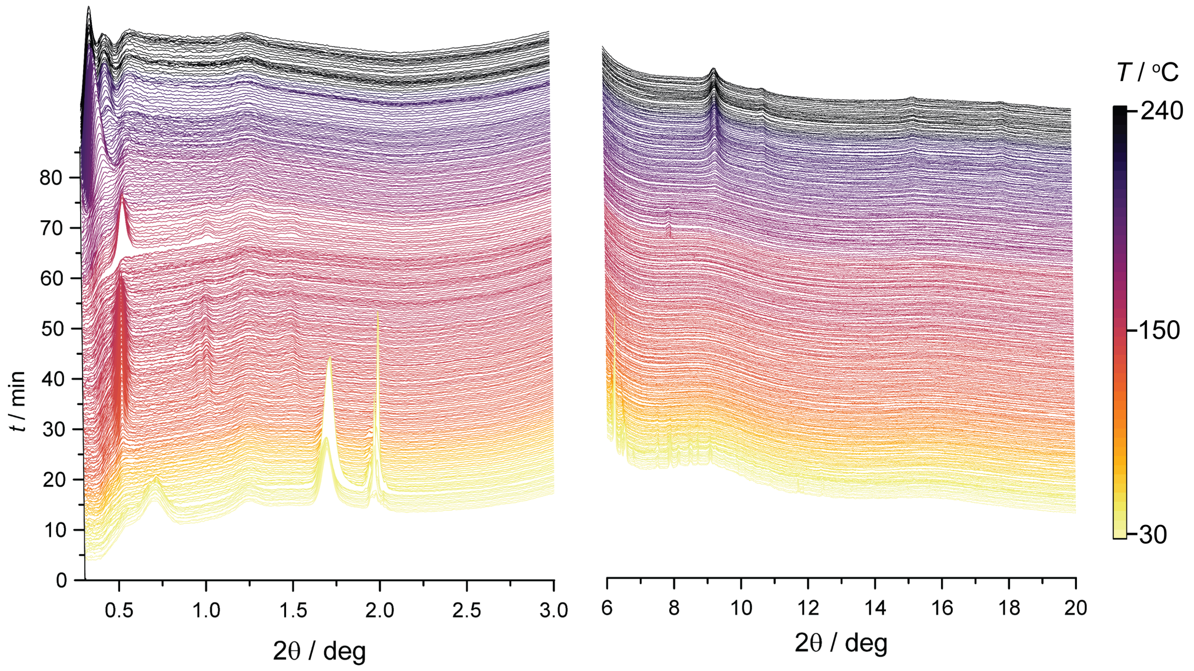

Figure S20. *In-situ* XRD data for the reaction of TDPA and Cu(OAc) in TOA solvent, showing the low- and high-angle regions. The reaction temperature is indicated by color.

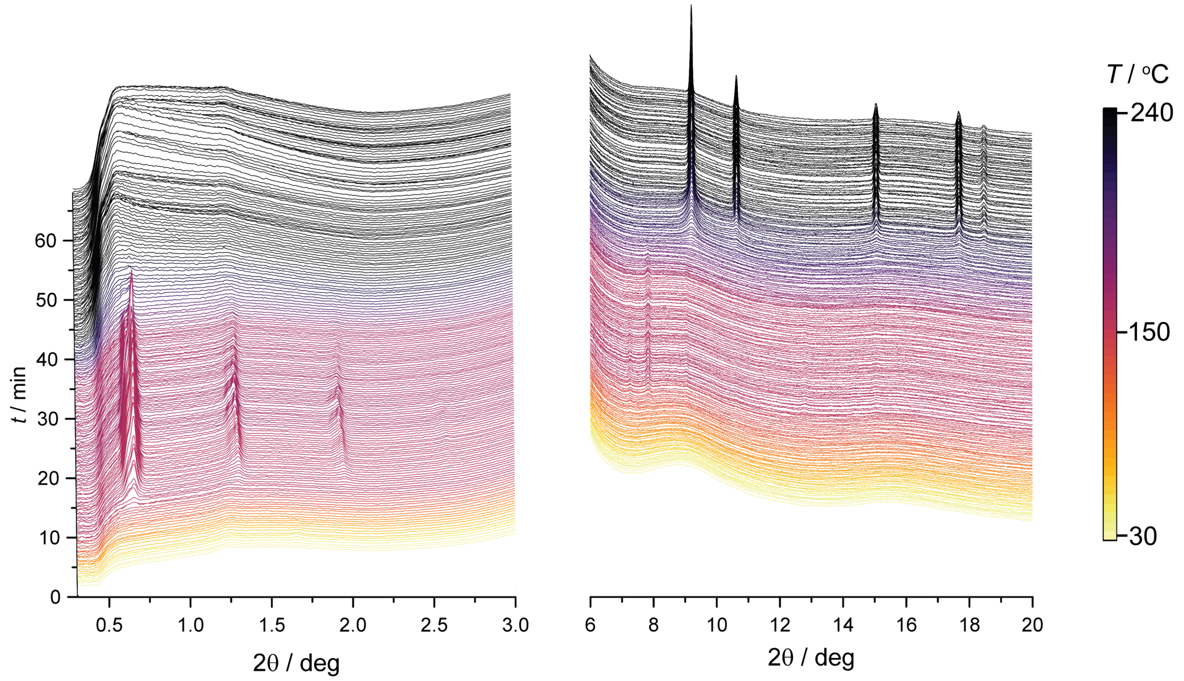

Figure S21. *In-situ* XRD data for the reaction of DPA and Cu(OAc) in TOA solvent, showing the low- and high-angle regions. The reaction temperature is indicated by color.

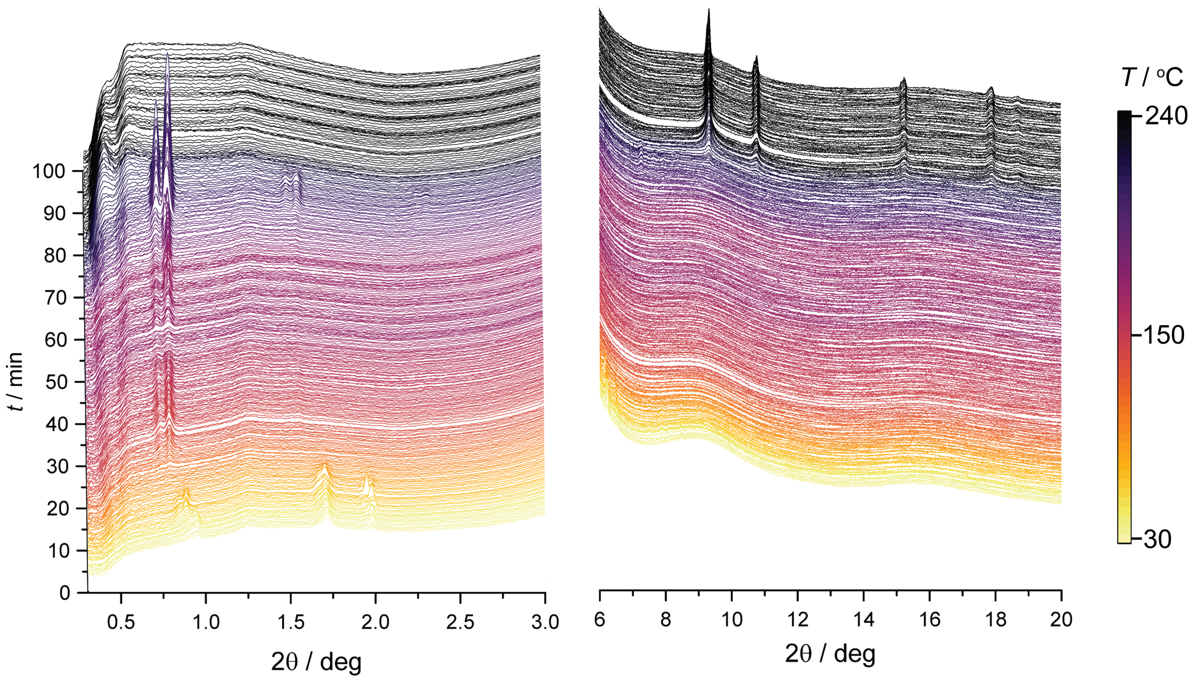

Figure S22. *In-situ* XRD data for the reaction of OPA and Cu(OAc) in TOA solvent, showing the low- and high-angle regions. The reaction temperature is indicated by color.

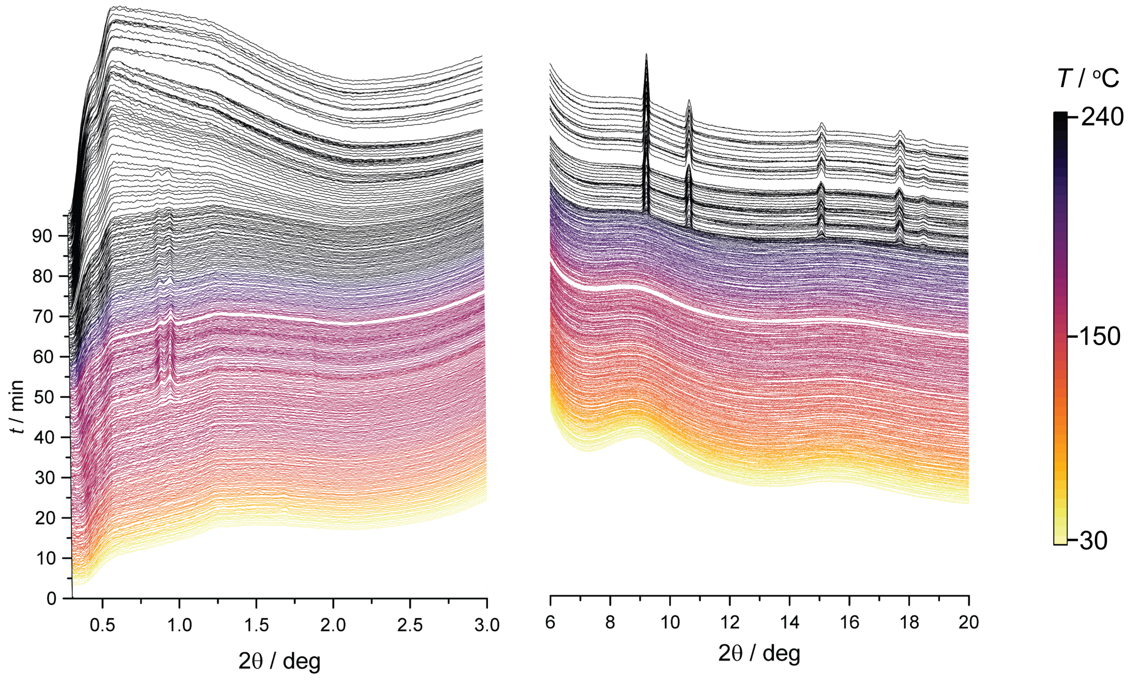

Figure S23. *In-situ* XRD data for the reaction of HPA and Cu(OAc) in TOA solvent, showing the low- and high-angle regions. The reaction temperature is indicated by color.

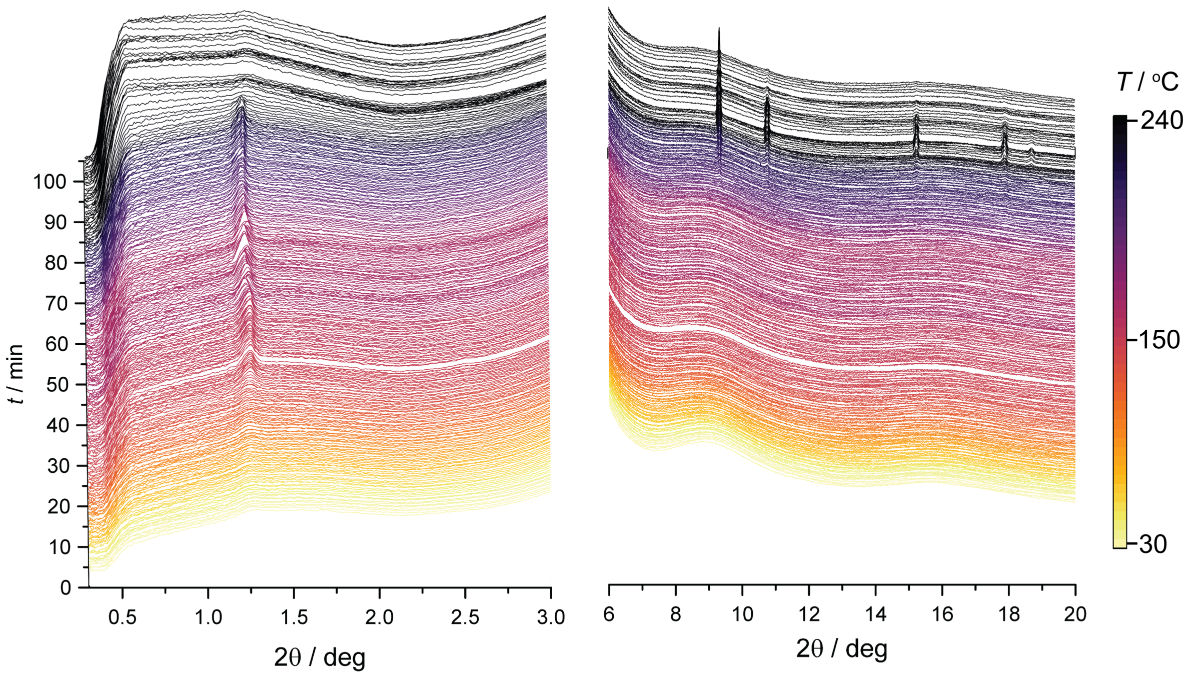

Figure S24. *In-situ* XRD data for the reaction of BPA and Cu(OAc) in TOA solvent, showing the low- and high-angle regions. The reaction temperature is indicated by color.

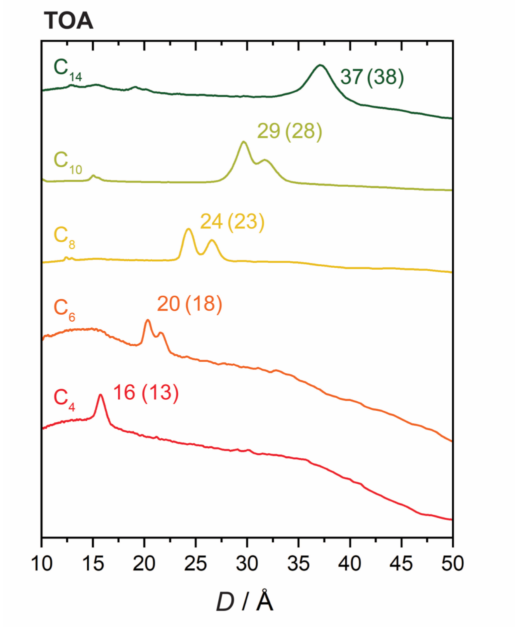

Figure S25. Selected *in-situ* XRD data taken from the temperature plateau at 150 °C, comparing the lamella spacing values when different-length phosphonic acid ligands are present. A clear dependence of the lamella spacing on the ligand length is observed, even in TOA solution. The values in brackets are the predicted spacings from molecular mechanics models.

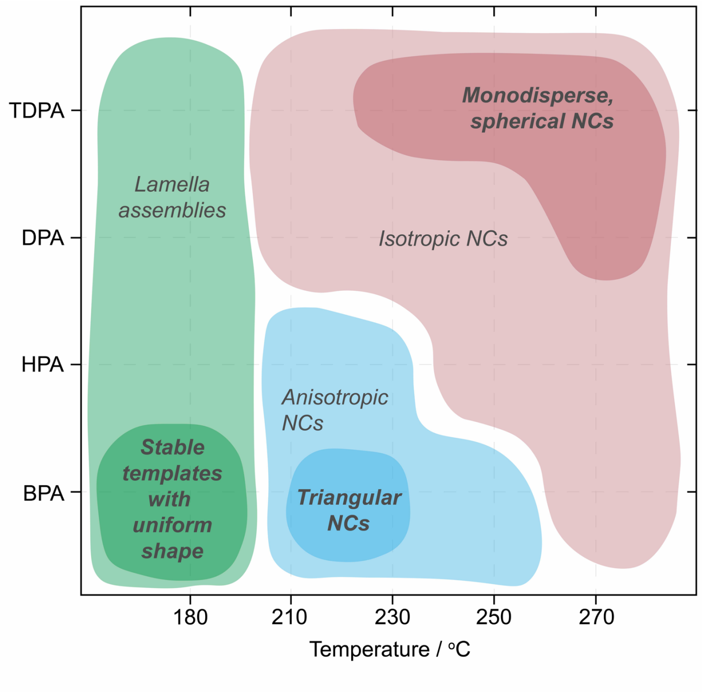

Figure S26. Schematic summary of products from reactions involving Cu(OAc) and phosphonic acid ligands of different length, isolated at different temperatures.

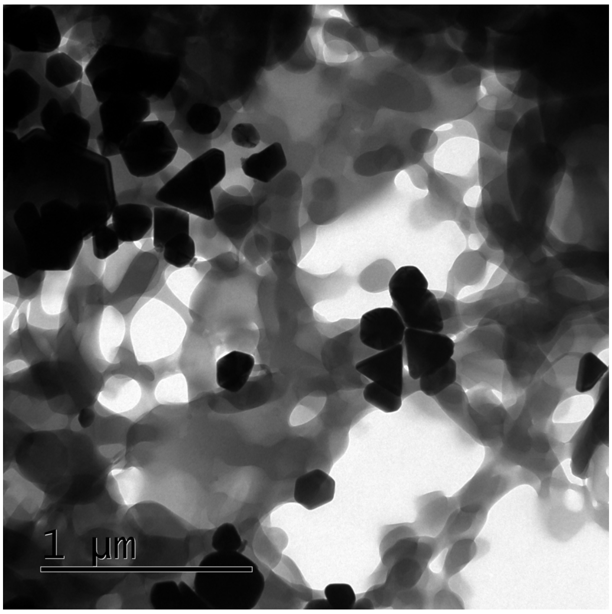

Figure S27. TEM image of the reaction product after heating a mixture of Cu(OAc), BPA and TOA at 180 °C for 48 hrs. A lot of unreacted lamella is present along with some Cu NC products, expressing a mixture of shapes, including some triangles and hexagons.

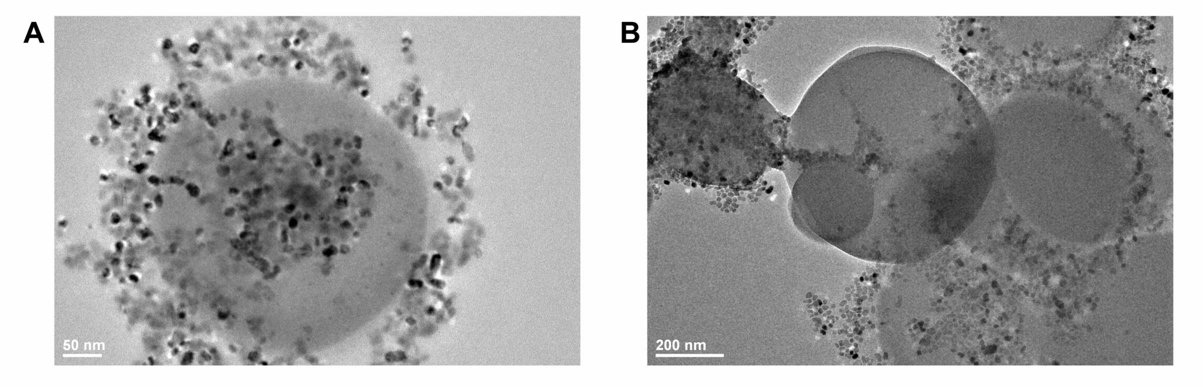

Figure S28. TEM images of the collected products after heating a mixture of Cu(OAc) and BPA in the solvent directly to T_2 (220 ºC) for 30 minutes, where the solvent is (**A**) TOA and (**B**) TOA + OLAM (3.3 v/v%). By skipping the plateau step at T_1, the NC yield is tremendously reduced to essentially zero. These results confirm the importance of building up a critical concentration of lamella mesostructure intermediates.

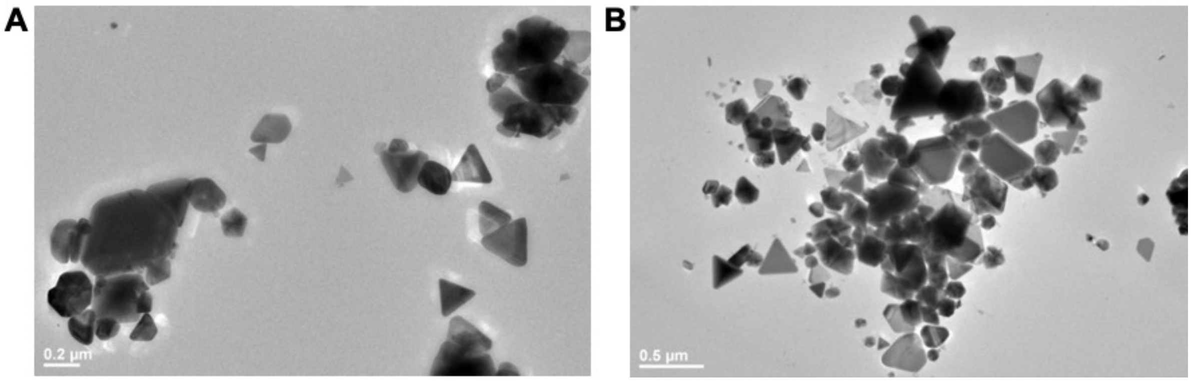

Figure S29. TEM images of the products obtained by pre-synthesizing the BPA lamellae and, upon washing, reacting them in (**A**) TOA at 220 ºC for 30 minutes and (**B**) TOA + OLAM (3.3 v/v%) at 220 ºC for 30 minutes, following the dropwise injection of OLAM. The BPA lamellae were synthesized by heating Cu(OAc), BPA and TOA + OLAM (3.3 v/v%) at 150 ºC for 30 minutes, followed by precipitation and solvent washing by centrifugation. The aim of this experiment was to remove any side-products from the initial reaction between Cu(OAc) and BPA during the lamella formation stage, before the NC formation.

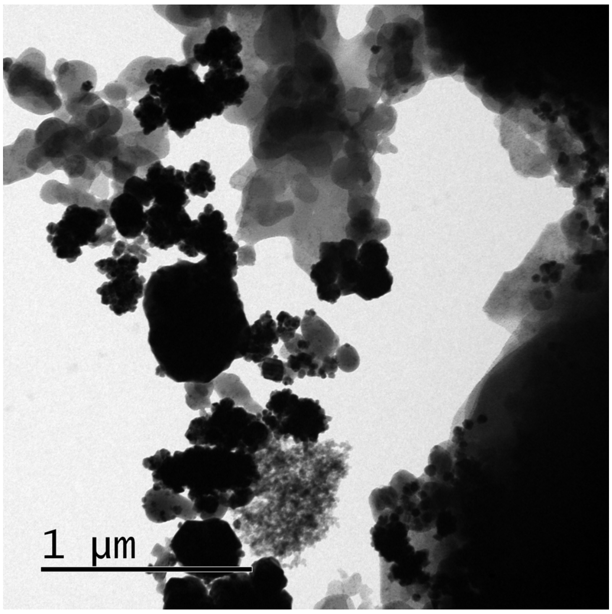

Figure S30. TEM image of the reaction product after heating a mixture of Cu(OAc), BPA and TOA at 150 ºC for 30 minutes to form the lamellae, cooling the mixture to 130 ºC and adding ascorbic acid at 130 ºC. The reaction was left to stir at 130 ºC for another 30 minutes. The aim was to reduce the lamella template at low temperature, which might result in a NC shape resembling the template. The large, irregularly shaped clusters evidence that the reduction is too rapid, or that temperature shock from the addition results in irregular NC growth.

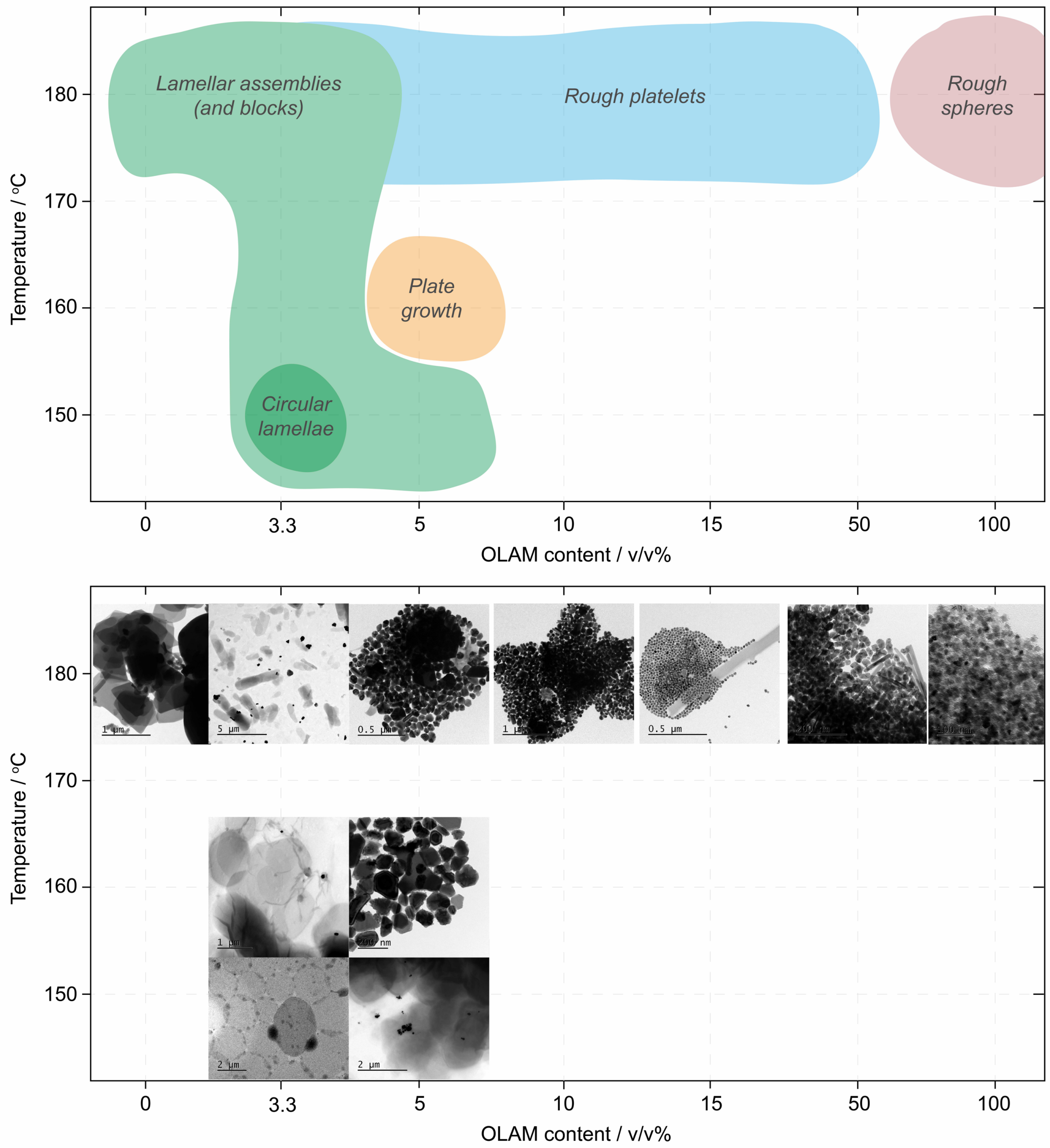

Figure S31. Exploring the effect of oleylamine (OLAM) content in the TOA solvent on the lamella intermediate, using BPA as the phosphonic acid ligand. **Top**: overview of the reaction space, showing the relationship between OLAM content and temperature. **Bottom**: corresponding TEM images of the products. Higher OLAM content caused reduction of the lamella intermediate into rough platelet / spherical NCs, even at 180 ºC. Below 5%, minimal lamella reduction was observed while the benefits of OLAM acting as a surfactant were still observed. Lowering the temperature of lamella formation (T_1) with 5% OLAM present resulted in 2D plate NCs at 160 ºC and no reduction at 150 ºC. Lowering the temperature of lamella formation (T_1) with 3.3% OLAM (1 mmol, 1:1 ratio with Cu(OAc)) present yielded the best results, especially at 150 ºC where homogeneous, circular lamellae where observed, nicely dispersed on the TEM grid.

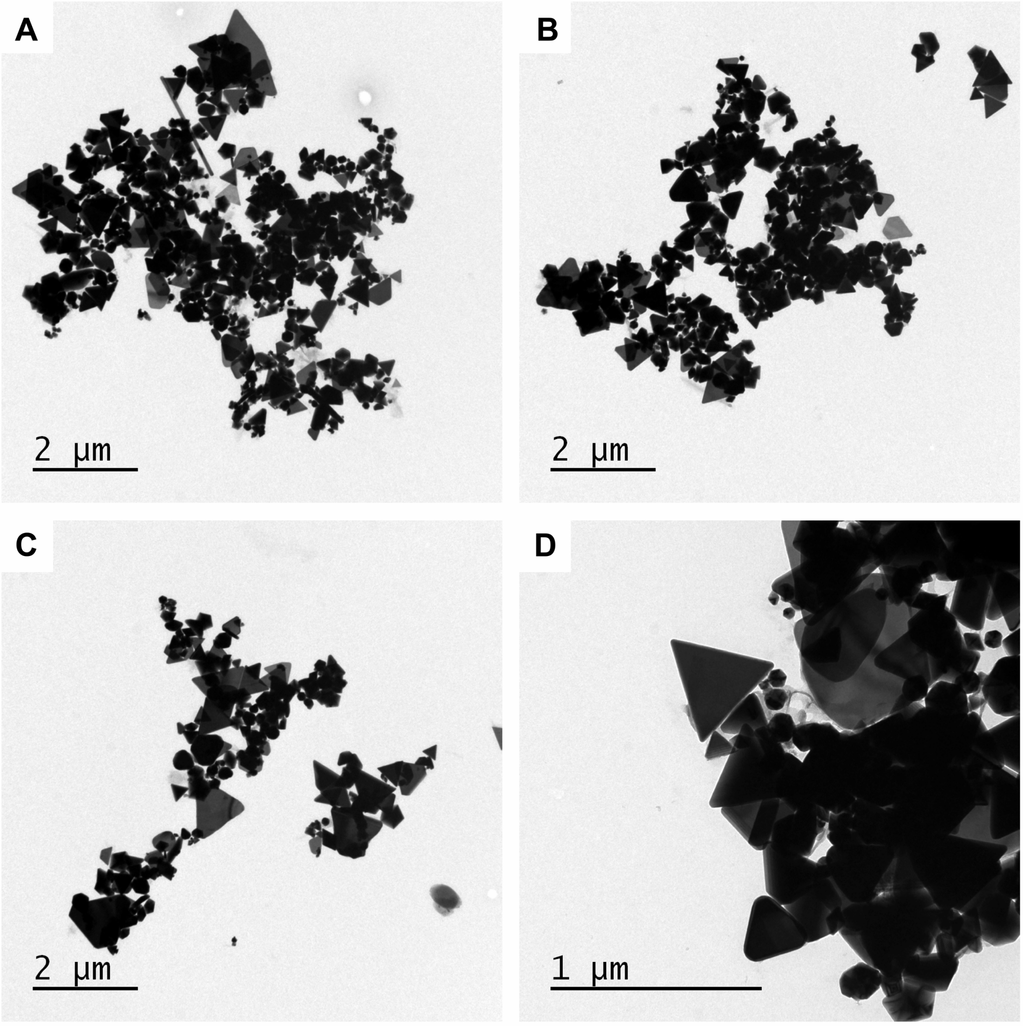

Figure S32. TEM images of the NC products obtained from a reaction containing Cu(OAc), BPA, TOA and 3.3 v/v% OLAM (1 mmol, 1 eq vs Cu), with a reaction temperature (T_2) of 220 ºC. While there are many blocks in the sample, the images evidence an abundance of anisotropic Cu NCs, with triangular NCs being the dominant product (quantitative analysis is given in **Figures S31** and **S32**). Only four images are shown here as representative examples, but many images were used for the size and shape statistics analysis shown below; only non-overlapping NCs were considered for the analysis.

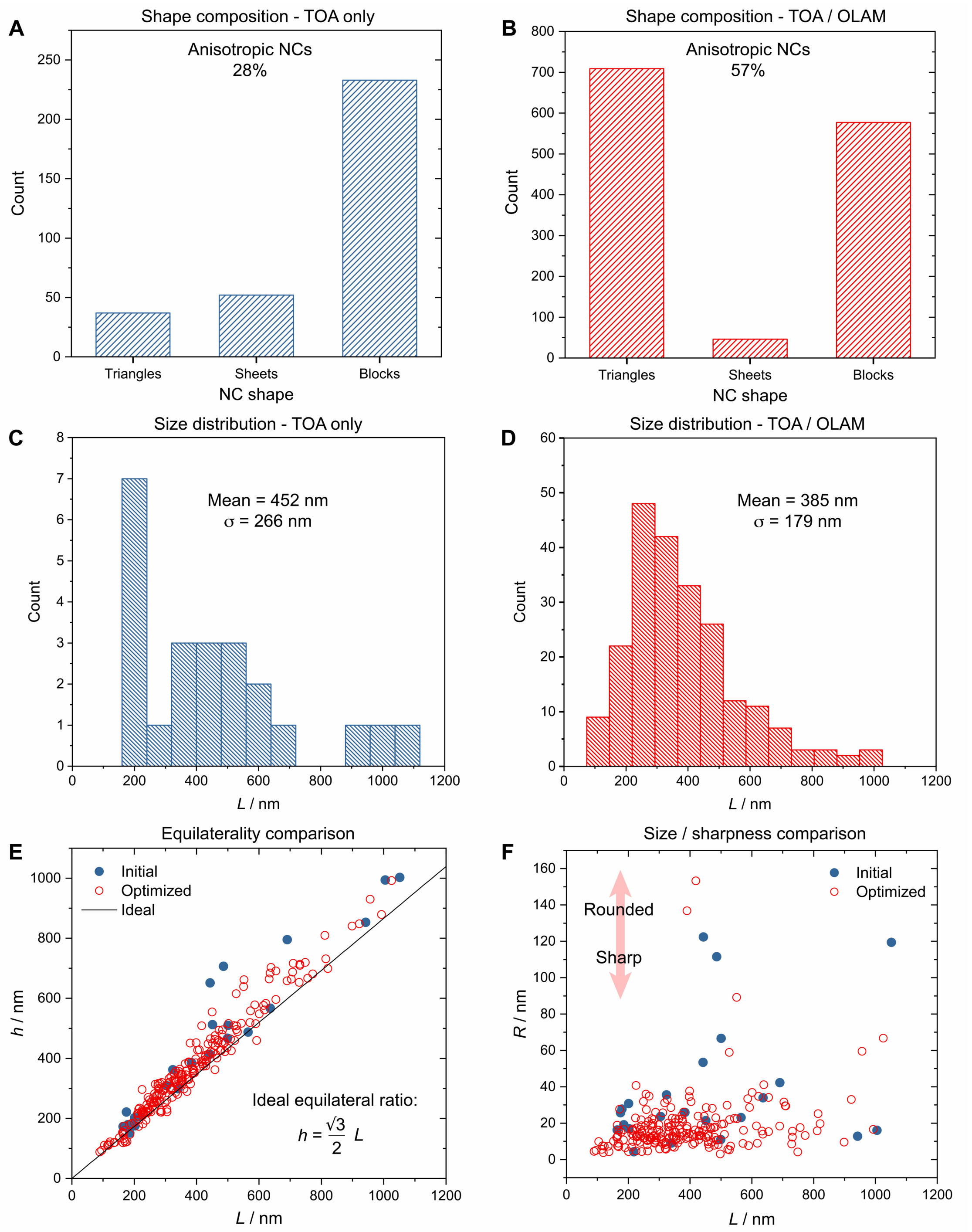

Figure S33. Analysis of anisotropic NC products obtained from BPA-derived lamellae, comparing the initial products from pure TOA solvent with the products from an optimized reaction involving OLAM in TOA. **A,B)** Histograms comparing the initial and optimized shape compositions, revealing the large increase in yield for anisotropic shapes, especially triangles, after optimization. **C,D)** Histograms comparing the initial and optimized size distributions, showing some improvement after optimization. **E)** Correlation between the triangle edge length, L, and the triangle height, h, for initial and optimized products, showing a high degree of equilaterality in both cases; the straight line shows the ideal height / length ratio, where $h = (\sqrt{3} / 2)L$. **F)** Correlation between L and the triangle sharpness, represented as the radius of curvature, R, showing a similar distribution before and after optimization. **Figure S32** illustrates the h, L and R parameters.

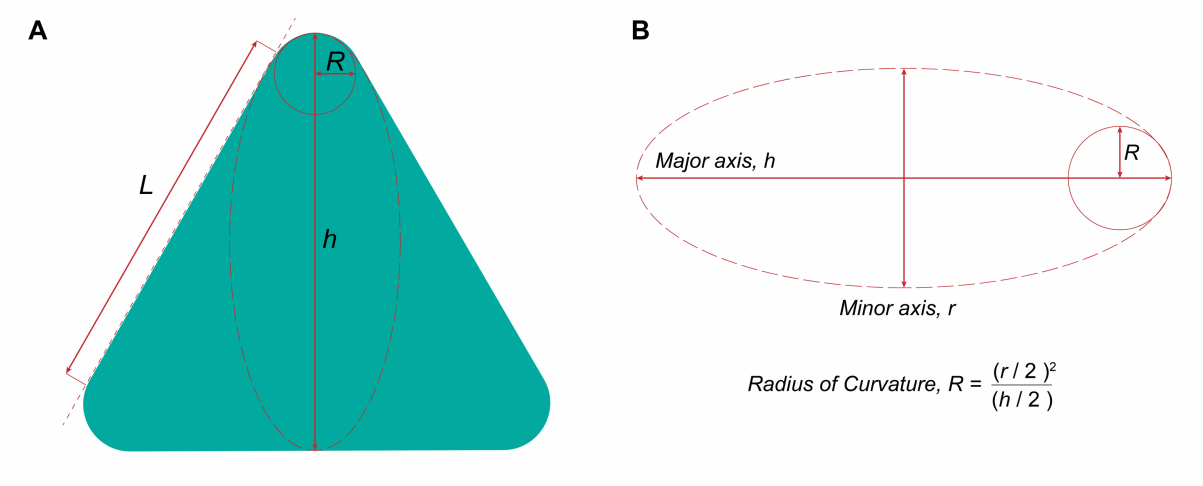

Figure S34. A) Schematic of the measured triangular NC parameters plotted in **Figure S31**, showing the edge length, *L*, the height, *h*, and the radius of curvature, *R*. **B)** Schematic showing how *R* is measured: an ellipse is fitted to the height of a triangle and its curvature at a corner; *R* is given by the equation, using measurements of the major and minor axes of the fitted ellipse.

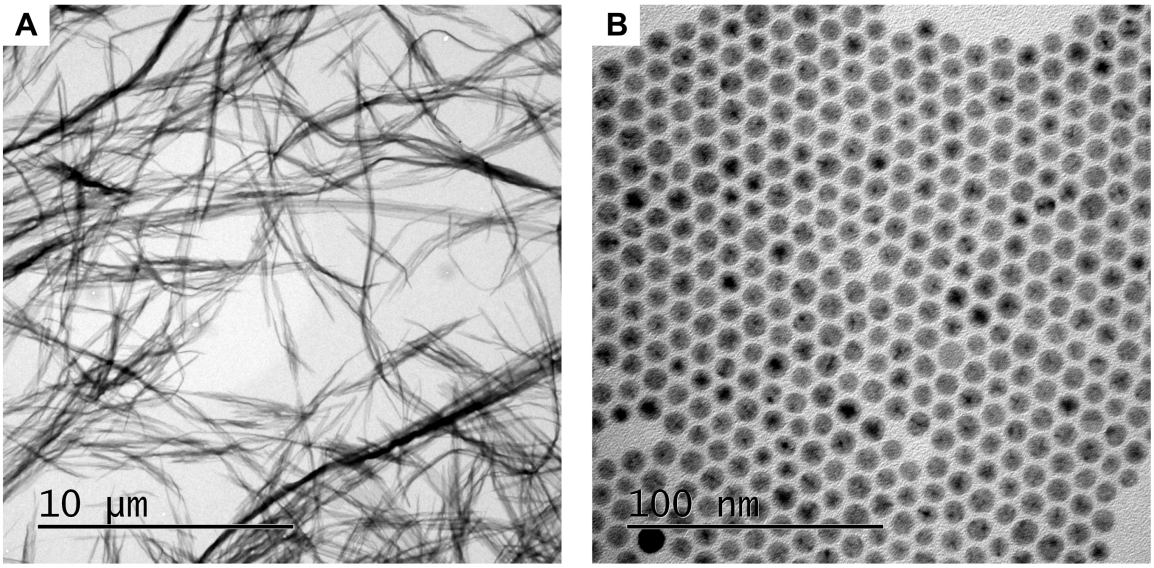

Figure S35. TEM images of (**A**) lamella intermediates and (**B**) the resulting Cu NCs from a reaction involving Cu(OAc), TDPA, TOA and OLAM. This result shows that OLAM itself is not responsible for the anisotropic shape of the Cu NCs, rather, it is the nature of the lamella intermediate that directs the growth, where only short-chain lamellae yield 2D Cu NCs.

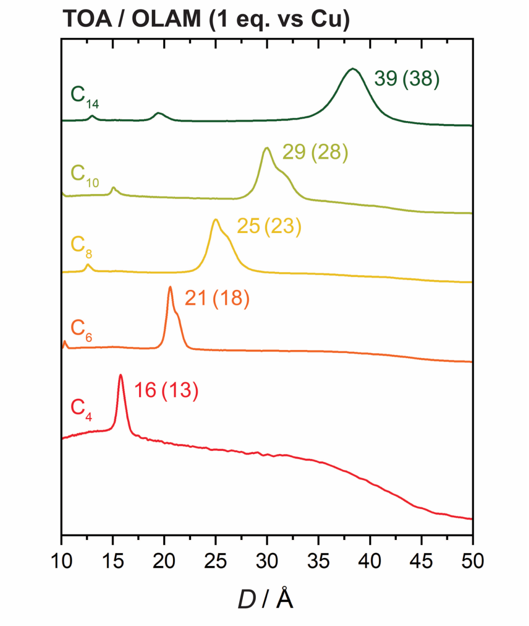

Figure S36. Selected *in-situ* XRD data taken from the temperature plateau at 150 ºC, comparing the lamella spacing values when different-length phosphonic acid ligands are present. A clear dependence of the lamella spacing on the ligand length is observed, even in TOA solutions containing OLAM. The values in brackets are the predicted spacings from molecular mechanics models.

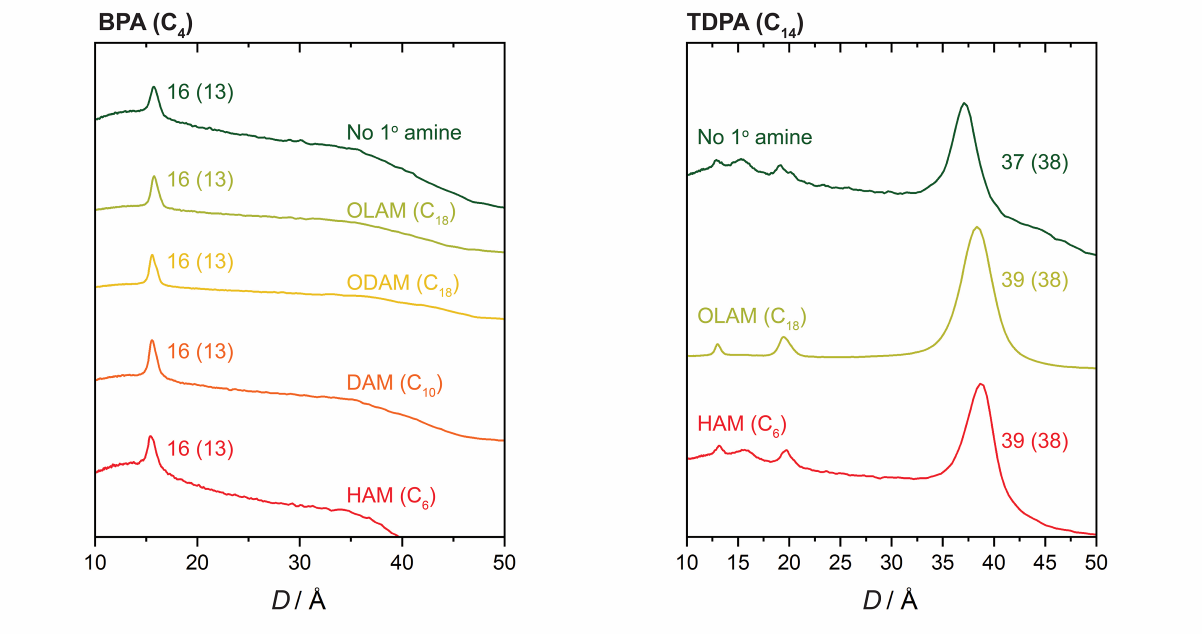

Figure S37. Selected *in-situ* XRD data taken from the temperature plateau at 150 ºC, comparing the lamella spacing values when different-length primary amines are present. A clear dependence of the lamella spacing on the length of the phosphonic acid is observed, for both BPA and TDPA reactions, regardless of the length of the added primary amine. The values in brackets are the predicted spacings from molecular mechanics models. These results rule out the inclusion of the primary amine (*e.g.* OLAM) in the lamella structure. OLAM = oleylamine, ODAM = octadecylamine, DAM = decylamine, HAM = hexylamine.

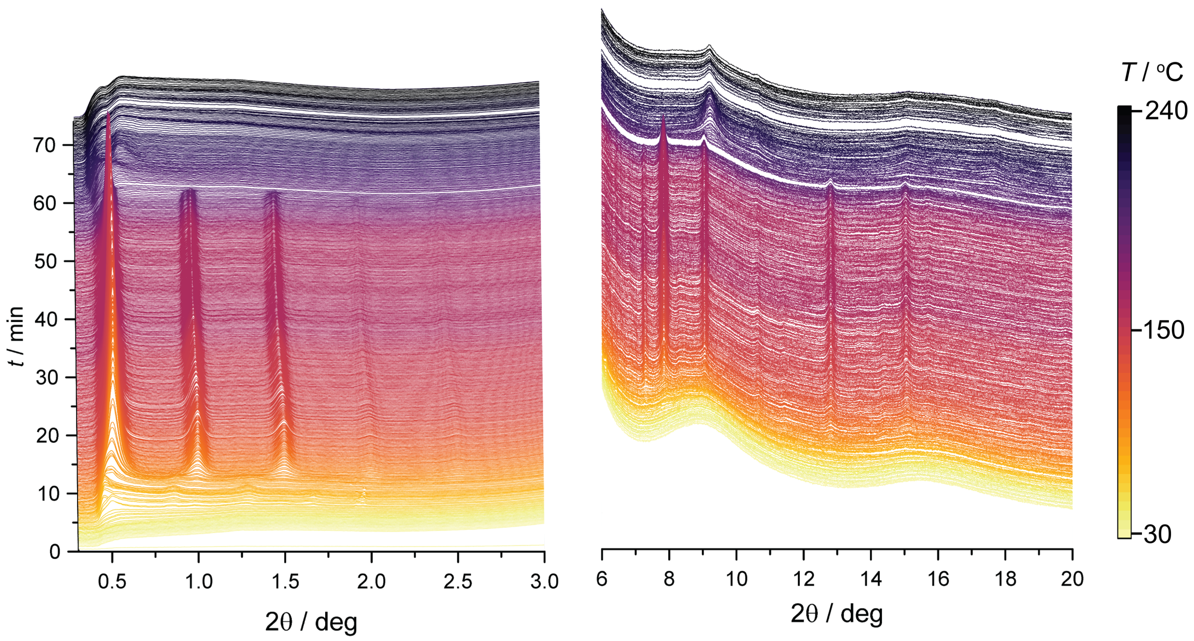

Figure S38. *In-situ* XRD data for the reaction of TDPA and Cu(OAc) in TOA / OLAM (3.3 v/v%, 1 mmol) solvent, showing the low- and high-angle regions. The reaction temperature is indicated by color. Note the large increase in signal-to-noise for the lamella peaks in comparison with **Figure S20**, due to the addition of OLAM improving the solubility of the intermediate.

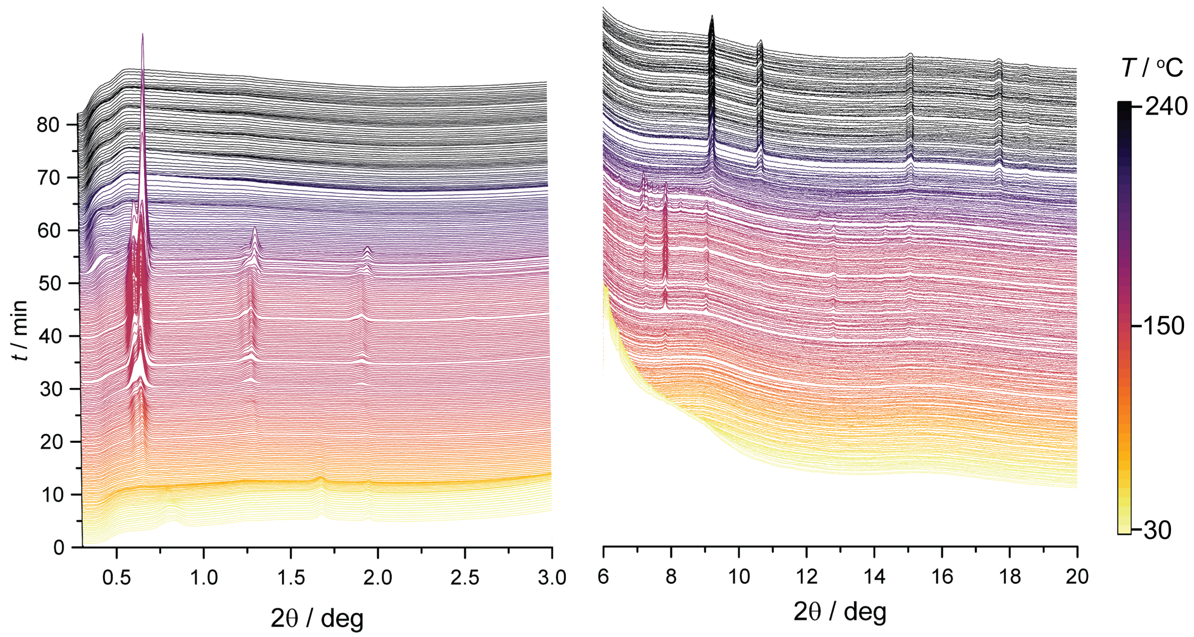

Figure S39. *In-situ* XRD data for the reaction of DPA and Cu(OAc) in TOA / OLAM (3.3 v/v%, 1 mmol) solvent, showing the low- and high-angle regions. The reaction temperature is indicated by color. Note the large increase in signal-to-noise for the lamella peaks in comparison with **Figure S21**, due to the addition of OLAM improving the solubility of the intermediate.

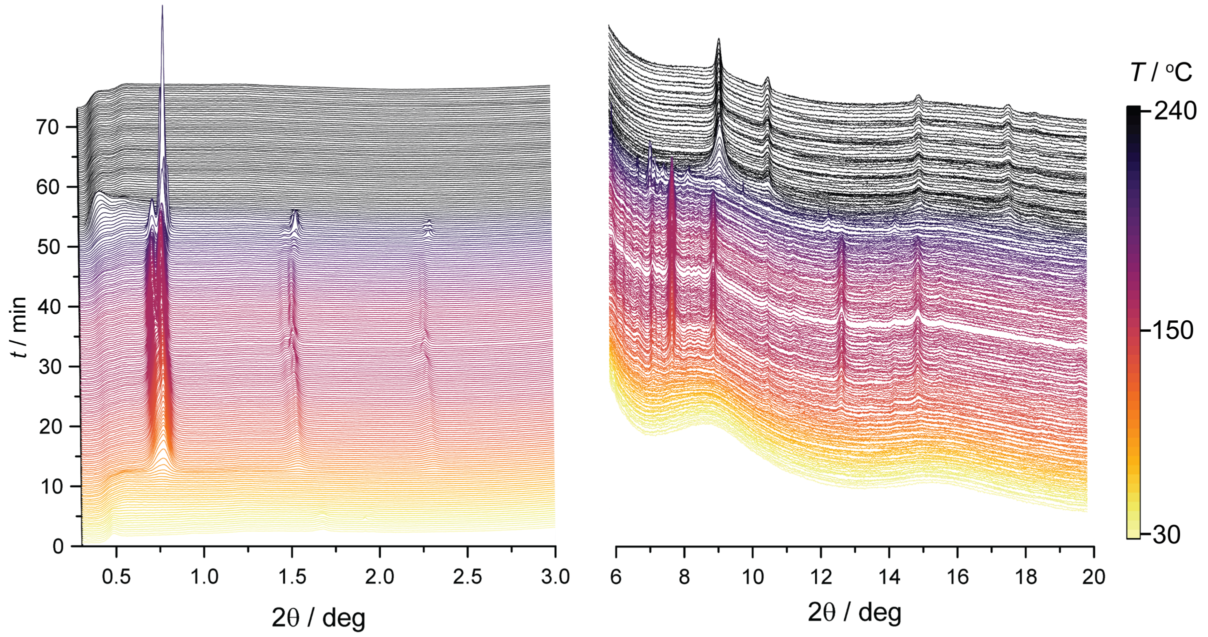

Figure S40. *In-situ* XRD data for the reaction of OPA and Cu(OAc) in TOA / OLAM (3.3 v/v%, 1 mmol) solvent, showing the low- and high-angle regions. The reaction temperature is indicated by color. Note the large increase in signal-to-noise for the lamella peaks in comparison with **Figure S22**, due to the addition of OLAM improving the solubility of the intermediate.

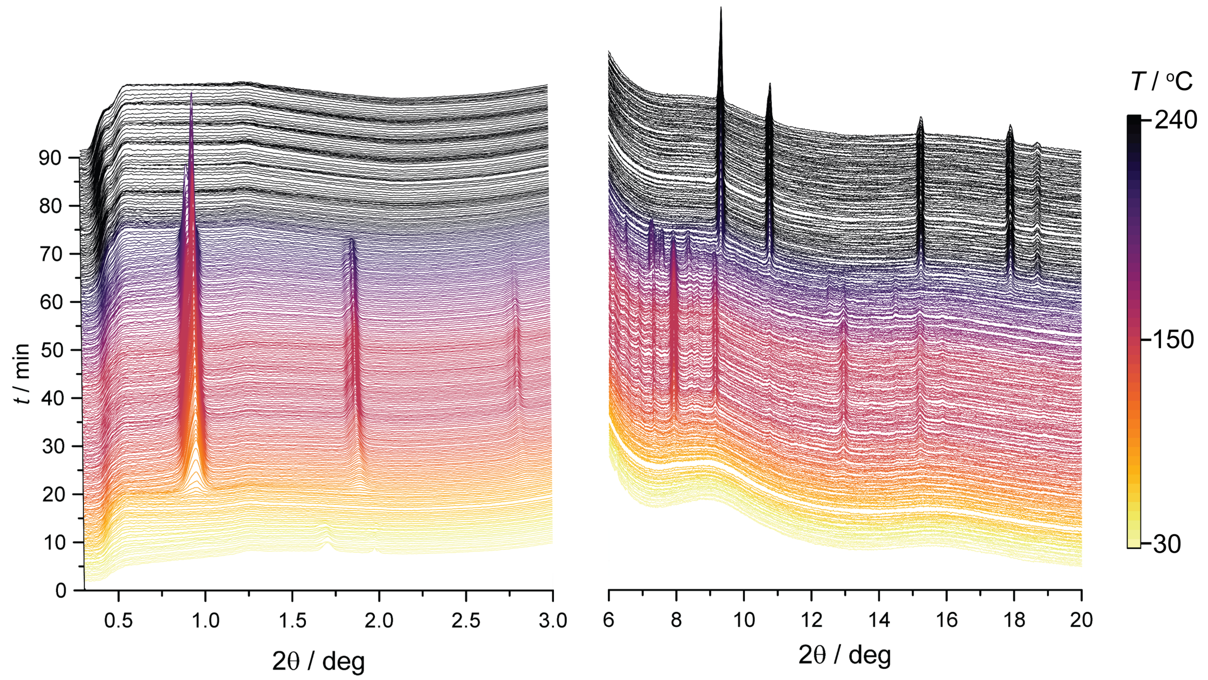

Figure S41. *In-situ* XRD data for the reaction of HPA and Cu(OAc) in TOA / OLAM (3.3 v/v%, 1 mmol) solvent, showing the low- and high-angle regions. The reaction temperature is indicated by color. Note the large increase in signal-to-noise for the lamella peaks in comparison with **Figure S23**, due to the addition of OLAM improving the solubility of the intermediate.

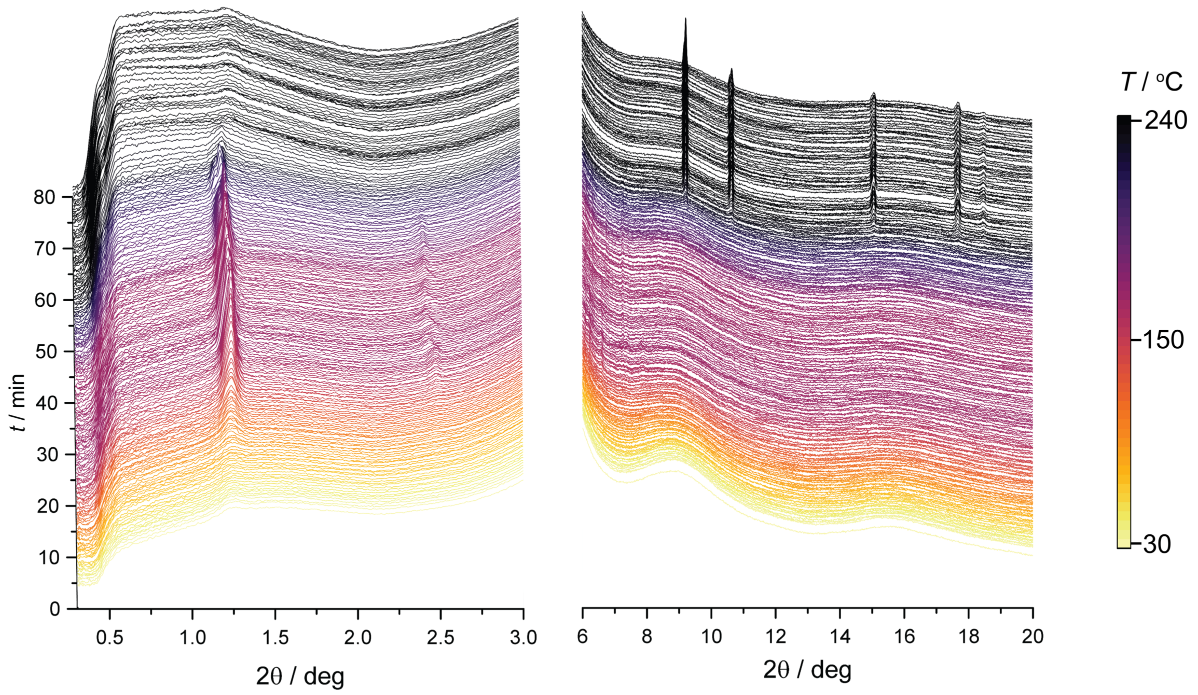

Figure S42. *In-situ* XRD data for the reaction of BPA and Cu(OAc) in TOA / OLAM (3.3 v/v%, 1 mmol) solvent, showing the low- and high-angle regions. The reaction temperature is indicated by color. Note the large increase in signal-to-noise for the lamella peaks in comparison with **Figure S24**, due to the addition of OLAM improving the solubility of the intermediate.

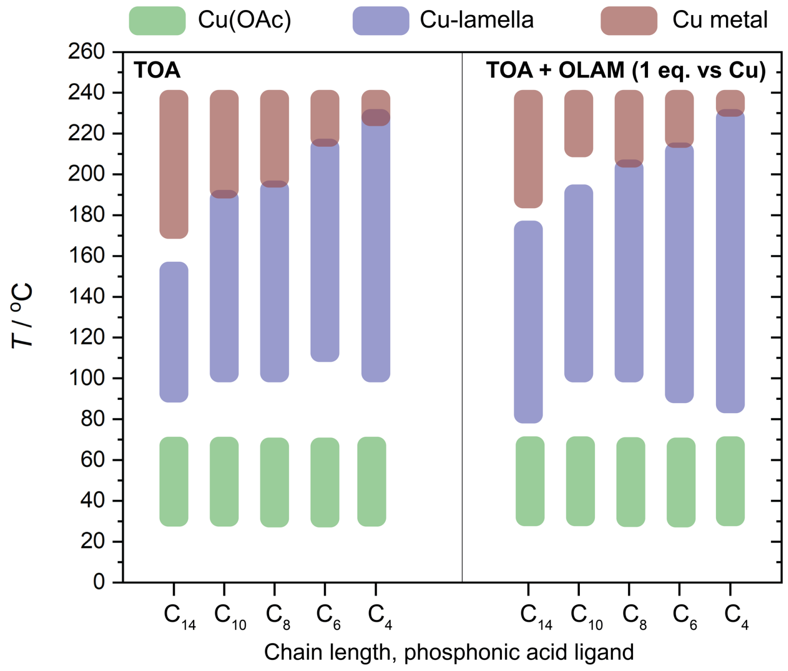

Figure S43. Summary of **Figures S36–S40**, showing the temperatures at which the different phases are observed by *in-situ* XRD, revealing the relationship between lamella stability and the phosphonic acid tail length. Identical observations are made in both pure TOA and TOA / OLAM solvents.

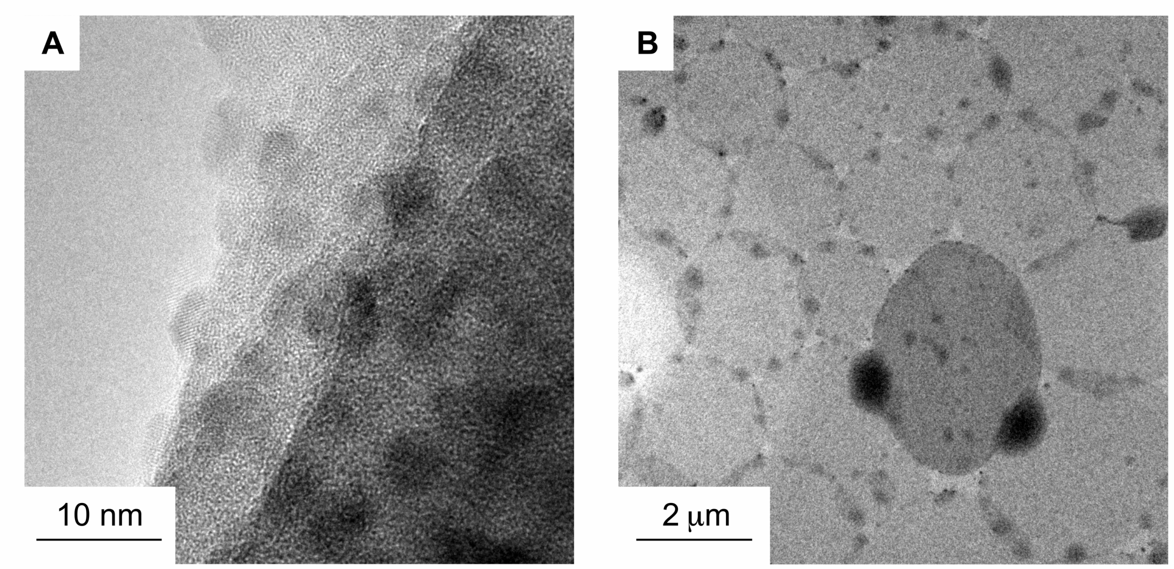

Figure S44. TEM images of lamella sheets, isolated at 150 °C from a reaction involving BPA and OLAM, showing **(A)** a high-resolution image of small, spherical Cu crystallites and **(B)** a low-resolution image of circular sheets. We note that the *in-situ* XRD data do not capture this early stage of crystallization because of the very low signal intensity that would arise from very broad peaks at low concentration in solution. Any diffraction from these small particles would be masked from the scattering from the solvent. However, the *in-situ* XAS data do reveal the buildup of Cu(0) species during the temperature plateau, which could be assigned to these crystallites.

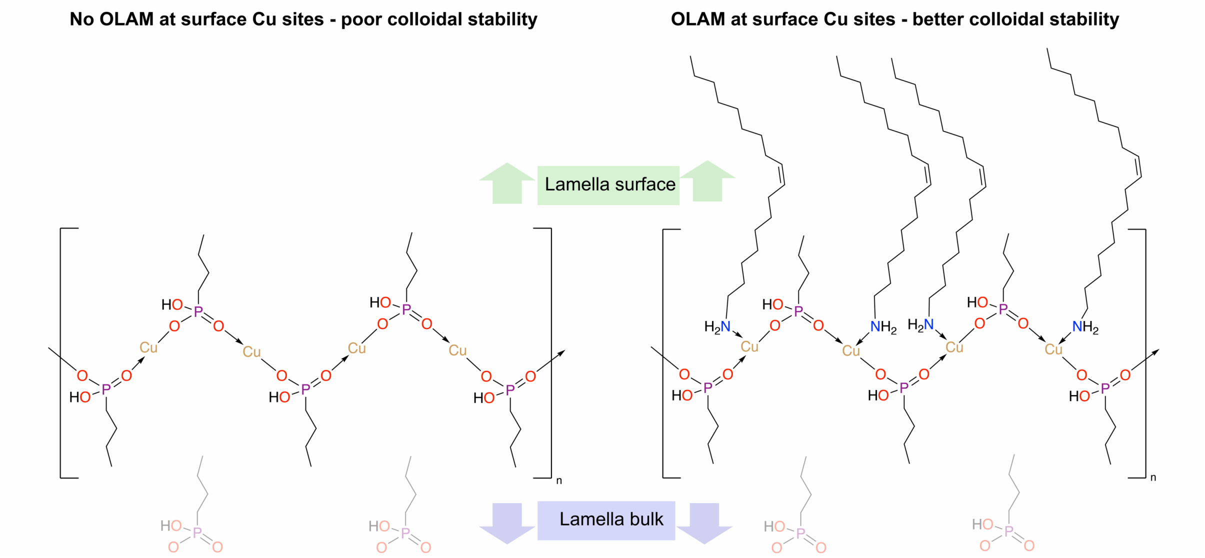

Figure S45. Proposed role of OLAM in the in the lamella-assisted synthesis of Cu NCs. As the XRD and XAS data provide evidence that the OLAM is not integrated into the lamella structure, the binding of OLAM to uncoordinated surface Cu sites is envisioned. As a result, the colloidal stability of the lamella mesostructures is improved, which results in the observed enhanced dispersibility (i.e. less aggregation in solution) and solubility. We propose that this enhanced homogeneity of the reaction medium containing the intermediates leads to an improvement in the NC product selectivity.

References

(1) Kieffer, J.; Wright, J. P. PyFAI: A Python Library for High Performance Azimuthal Integration on GPU. *Powder Diffr.* **2013**, *28*, S339–S350

(2) Mantella, V.; Strach, M.; Frank, K.; Pankhurst, J. R.; Stoian, D.; Gadiyar, C.; Nickel, B.; Buonsanti, R. Polymer Lamellae as Reaction Intermediates in the Formation of Copper Nanospheres as Evidenced by In Situ X-ray Studies. *Angew. Chem. Int. Ed.* **2020**, *59*, 11627–11633

(3) Strach, M.; Mantella, V.; Pankhurst, J. R.; Iyengar, P.; Loiudice, A.; Das, S.; Corminboeuf, C.; van Beek, W.; Buonsanti, R. Insights into Reaction Intermediates to Predict Synthetic Pathways for Shape-Controlled Metal Nanocrystals. *J. Am. Chem. Soc.* **2019**, *141*, 16312–16322

(4) Figueroa, S. J. A.; Prestipino, C. PrestoPronto: A Code Devoted to Handling Large Data Sets. *J. Phys.: Conf. Ser.* **2016**, *712*, 012012

(5) Ravel, B.; Newville, M. *ATHENA* , *ARTEMIS* , *HEPHAESTUS* : Data Analysis for X-Ray Absorption Spectroscopy Using *IFEFFIT*. *J Synchrotron Rad* **2005**, *12*, 537–541

(6) de Juan, A.; Jaumot, J.; Tauler, R. Multivariate Curve Resolution (MCR). Solving the Mixture Analysis Problem. *Anal. Methods* **2014**, *6*, 4964–4976

(7) Jaumot, J.; Gargallo, R.; de Juan, A.; Tauler, R. A Graphical User-Friendly Interface for MCR-ALS: A New Tool for Multivariate Curve Resolution in MATLAB. *Chemometrics and Intelligent Laboratory Systems* **2005**, *76*, 101–110

(8) Voronov, A.; Urakawa, A.; Beek, W. van; Tsakoumis, N. E.; Emerich, H.; Rønning, M. Multivariate Curve Resolution Applied to in Situ X-Ray Absorption Spectroscopy Data: An Efficient Tool for Data Processing and Analysis. *Analytica Chimica Acta* **2014**, *840*, 20–27

(9) Available free of charge at https://mcrals.wordpress.com/

(10) Sun, S.; Murray, C. B. Synthesis of Monodisperse Cobalt Nanocrystals and Their Assembly into Magnetic Superlattices (Invited). *Journal of Applied Physics* **1999**, *85*, 4325–4330

(11) Yamamoto, M.; Nakamoto, M. Novel Preparation of Monodispersed Silver Nanoparticles via Amine Adducts Derived from Insoluble Silver Myristate in Tertiary Alkylamine. *J. Mater. Chem.* **2003**, *13*, 2064

(12) Pan, A.; He, B.; Fan, X.; Liu, Z.; Urban, J. J.; Alivisatos, A. P.; He, L.; Liu, Y. Insight into the Ligand-Mediated Synthesis of Colloidal $CsPbBr_3$ Perovskite Nanocrystals: The Role of Organic Acid, Base, and Cesium Precursors. *ACS Nano* **2016**, *10*, 7943–7954

(13) Jang, D. M.; Kim, D. H.; Park, K.; Park, J.; Lee, J. W.; Song, J. K. Ultrasound Synthesis of Lead Halide Perovskite Nanocrystals. *J. Mater. Chem. C* **2016**, *4*, 10625–10629
